# Mucosal vaccine-induced cross-reactive CD8^+^ T cells protect against SARS-CoV-2 XBB.1.5 respiratory tract infection

**DOI:** 10.1038/s41590-024-01743-x

**Published:** 2024-02-09

**Authors:** Baoling Ying, Tamarand L. Darling, Pritesh Desai, Chieh-Yu Liang, Igor P. Dmitriev, Nadia Soudani, Traci Bricker, Elena A. Kashentseva, Houda Harastani, Saravanan Raju, Meizi Liu, Aaron G. Schmidt, David T. Curiel, Adrianus C. M. Boon, Michael S. Diamond

**Affiliations:** 1 Department of Medicine, Washington University School of Medicine, St. Louis, MO USA; 2 Department of Pathology & Immunology, Washington University School of Medicine, St. Louis, MO USA; 3 Department of Radiation Oncology, Washington University School of Medicine, St. Louis, MO USA; 4 Ragon Institute of Massachusetts General Hospital, Massachusetts Institute of Technology and Harvard University, Cambridge, MA USA; 5 Department of Microbiology, Harvard Medical School, Boston, MA USA; 6 Department of Molecular Microbiology, Washington University School of Medicine, St. Louis, MO USA; 7 Andrew M. and Jane M. Bursky Center for Human Immunology and Immunotherapy Programs, Washington University School of Medicine, St. Louis, MO USA; 8 Center for Vaccines and Immunity to Microbial Pathogens, Washington University School of Medicine, St. Louis, MO USA

**Keywords:** RNA vaccines, Viral infection, Immunological memory, SARS-CoV-2

## Abstract

A nasally delivered chimpanzee adenoviral-vectored severe acute respiratory syndrome coronavirus 2 (SARS-CoV-2) vaccine (ChAd-SARS-CoV-2-S) is currently used in India (iNCOVACC). Here, we update this vaccine by creating ChAd-SARS-CoV-2-BA.5-S, which encodes a prefusion-stabilized BA.5 spike protein. Whereas serum neutralizing antibody responses induced by monovalent or bivalent adenoviral vaccines were poor against the antigenically distant XBB.1.5 strain and insufficient to protect in passive transfer experiments, mucosal antibody and cross-reactive memory T cell responses were robust, and protection was evident against WA1/2020 D614G and Omicron variants BQ.1.1 and XBB.1.5 in mice and hamsters. However, depletion of memory CD8^+^ T cells before XBB.1.5 challenge resulted in loss of protection against upper and lower respiratory tract infection. Thus, nasally delivered vaccines stimulate mucosal immunity against emerging SARS-CoV-2 strains, and cross-reactive memory CD8^+^ T cells mediate protection against lung infection by antigenically distant strains in the setting of low serum levels of cross-reactive neutralizing antibodies.

## Abstract (web summary)

Here, the authors enhance their nasally delivered chimpanzee adenoviral-vectored SARS-CoV-2 vaccine with an Omicron-matched vaccine (ChAd-SARS-CoV-2-BA.5-S) that stimulates mucosal immunity in mice and hamsters and shows cross-reactive CD8^+^ memory T cell-driven protection against antigenically distant strains.

## Main

In response to the coronavirus disease 2019 (COVID-19) pandemic, multiple vaccines targeting the severe acute respiratory syndrome coronavirus 2 (SARS-CoV-2) spike (S) protein were developed and deployed. Most approved SARS-CoV-2 vaccines are delivered intramuscularly and have been highly effective (up to 95%) against early pandemic strains at preventing symptomatic infection, serious illness and death^1–4^. As successive variants have emerged, vaccine efficacy has declined such that protection against symptomatic infection by Omicron lineage strains is now less than 50% (ref. ^5^) due to the increasing immune evasion properties associated with large numbers of amino acid substitutions and deletions in the S protein compared to ancestral SARS-CoV-2 strains^6–10^.

Currently approved vaccine boosters have low efficacy against transmission of Omicron lineage viruses because of a poor capacity to induce mucosal immunity^11–13^. The development of oral, nasal or inhaled vaccines against SARS-CoV-2 is one strategy to induce mucosal responses that can better protect against infection and transmission of SARS-CoV-2 variants. Globally, there are approximately 100 mucosal vaccines against SARS-CoV-2 in development^14^, and preclinical studies have shown that nasally delivered vaccines targeting the Wuhan-1 S protein induce mucosal immunity and protect against infection by strains from early in the pandemic^15–21^. Two nasally delivered, adenoviral-vectored COVID-19 vaccines (iNCOVACC (chimpanzee adenoviral (ChAd)-SARS-CoV-2-S) and Convidecia Air (human Ad5-nCoV-inhaled)) targeting the S protein of the Wuhan-1 strain were approved in late 2022 in India and China, respectively, for use as primary or booster immunizations. Nonetheless, data on the efficacy of these nasally delivered vaccines against transmission^22,23^ or circulating antigen-shifted Omicron strains are absent.

Here, we present an updated ChAd-vectored vaccine (ChAd-SARS-CoV-2-BA.5-S) encoding a prefusion-stabilized S protein of the BA.5 strain. We evaluated the systemic and mucosal immune responses of intranasally (i.n.) delivered, single-dose monovalent or bivalent vaccines and their protective activity against ancestral WA1/2020 D614G and two antigenically shifted Omicron strains (BQ.1.1 and XBB.1.5) in susceptible K18-hACE2 transgenic mice and Syrian hamsters.

## Results

### Bivalent ChAd vaccine induces broadly reactive antibody responses

**Fig. 1:**
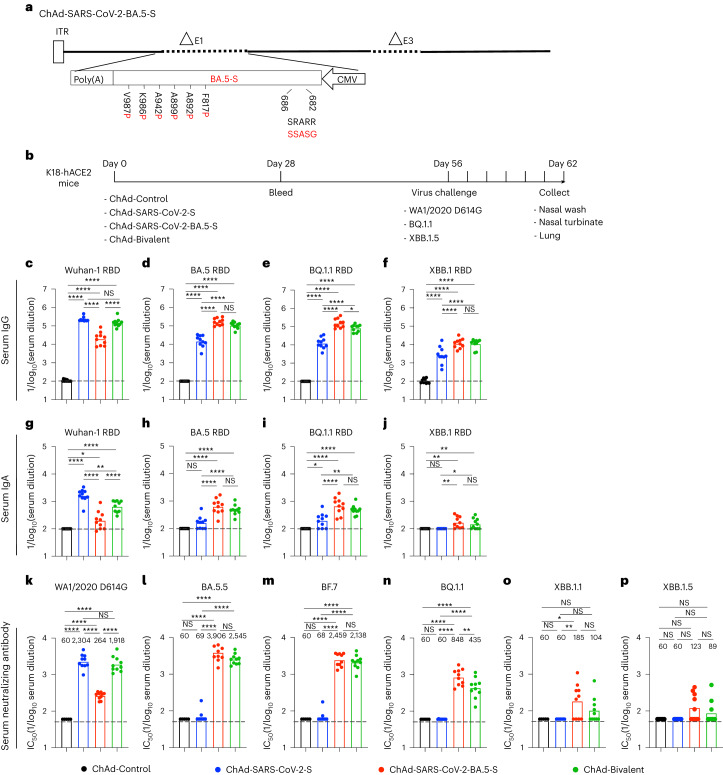
Antibody responses in the serum of K18-hACE2 mice after ChAd-vectored vaccine immunization. **a**, Diagram of the ChAd-SARS-CoV-2-BA.5-S vaccine encoding the Omicron BA.5 S protein with the indicated furin cleavage site and six proline substitutions, with substitutions in the BA.5 S protein shown in red; the positions of the residues correspond to the ancestral SARS-CoV-2 Wuhan-1 S protein; ITR, inverted terminal repeats; CMV, cytomegalovirus. **b**, Scheme of immunizations, blood collection and virus challenge. **c**–**p**, Cohorts of 7- to 9-week-old female K18-hACE2 mice were immunized i.n. with ChAd-Control, ChAd-SARS-CoV-2-S, ChAd-SARS-CoV-2-BA.5-S or bivalent ChAd vaccine. Sera were collected 4 weeks after immunization, and serum RBD-specific IgG (**c**–**f**) and IgA (**g**–**j**) levels (*n* = 10, two experiments) and neutralizing antibody titers (**k**–**p**; *n* = 5, 10, 10, 10 and 10 (left to right), two experiments) against the indicated authentic SARS-CoV-2 strains were determined. Boxes illustrate geometric mean values, and dotted lines show the LOD. Data were analyzed by one-way analysis of variance (ANOVA) with a Tukey’s post hoc test (**c**–**j**) or Dunnett’s post hoc test (**k**–**p**); *P < 0.05, **P < 0.01 and ****P < 0.0001; IC_50_, half-maximal inhibitory concentration; NS, not significant. Source data

We updated our replication-incompetent ChAd-vectored vaccine so that it encodes a prefusion-stabilized, full-length S protein of the BA.5 strain (ChAd-SARS-CoV-2-BA.5-S; GenBank: QJQ84760) with furin cleavage site substitutions (RRARS > GSASS) to enhance cell surface expression (Fig. 1a). We compared immune responses to the monovalent (ChAd-SARS-CoV-2-S or ChAd-SARS-CoV-2-BA.5-S) or bivalent (1:1 mixture of ChAd-SARS-CoV-2-S and ChAd-SARS-CoV-2-BA.5-S) vaccines by i.n. immunizing cohorts of 7-week-old female K18-hACE2 mice once with 2 × 10^9^ virus particles (Fig. 1b). Serum samples were collected 4 weeks later, and IgG and IgA responses to Wuhan-1, BA.5, BQ.1.1 and XBB.1 receptor-binding domain (RBD) proteins were measured (Fig. 1c–j). Whereas the ChAd-Control vaccine did not generate RBD-specific antibodies, the mono- and bivalent ChAd-SARS-CoV-2 vaccines induced IgG responses against all RBD proteins. Mice immunized with ChAd-SARS-CoV-2-S had high serum IgG titers against the RBD of Wuhan-1 but approximately 15- to 52-fold reductions against BA.5, BQ.1.1 and XBB.1 (Fig. 1c–f). In comparison, mice immunized with ChAd-SARS-CoV-2-BA.5-S showed high IgG titers against the RBD of BA.5 and BQ.1.1, with 8- to 12-fold lower titers against the RBD of Wuhan-1 and XBB.1 (Fig. 1c–f). A distinct pattern was observed in mice immunized with bivalent vaccine, which induced comparably high IgG titers against the RBD of Wuhan-1, BA.5 and BQ.1.1 and approximately tenfold lower IgG titers against the RBD of XBB.1 (Fig. 1c–f). A similar pattern of binding activity against the RBD of different SARS-CoV-2 strains was observed for serum IgA (Fig. 1g–j).

**Extended Data Fig. 1:**
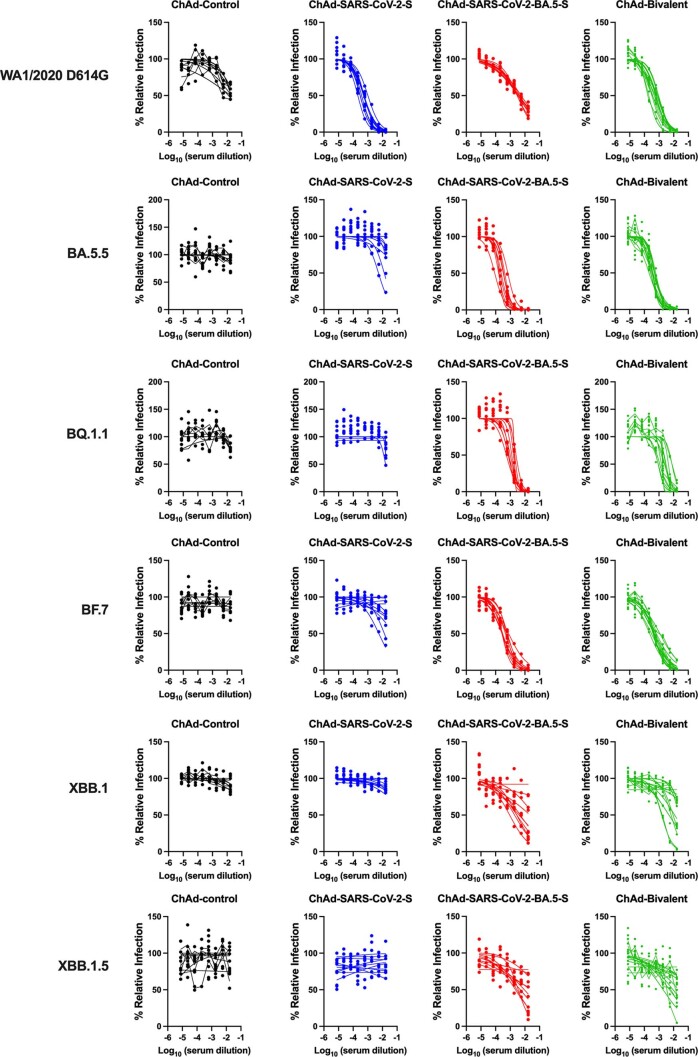
Serum neutralization of SARS-CoV-2 strains. 7 to 9-week-old female K18-hACE2 transgenic mice were immunized with ChAd-Control, ChAd-SARS-CoV-2-S, ChAd-SARS-CoV-2-BA.5-S, or ChAd-bivalent vaccine, and sera were collected four weeks after immunization. Serum neutralization curves against the indicated virus strains are shown for each vaccine (n = 8–10 per group). Each point represents the mean of two technical replicates. Source data

**Extended Data Fig. 2:**
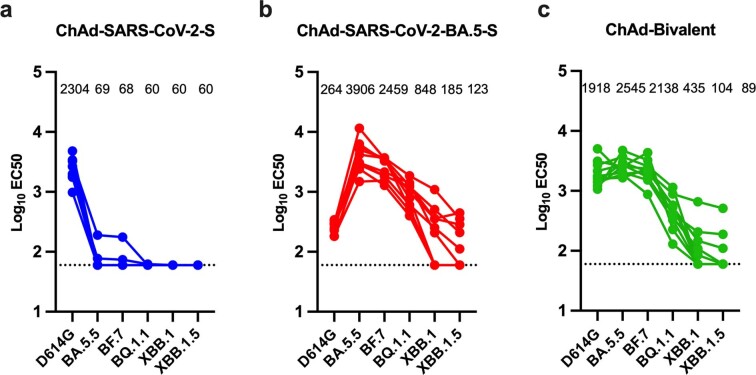
Comparison of serum neutralizing activity induced by ChAd vaccines against SARS-CoV-2 strains. Comparison of neutralizing activity is shown for serum samples from immunized mice against WA1/2020 D614G, BA.5, BF.7, BQ.1.1, XBB.1.1 and XBB.1.5. Results are from experiments performed in Fig. 1k–p. Geometric mean neutralization titers (GMT) are shown above each graph, and dotted lines represent the LOD. Solid lines connect data points from the same serum sample across strains. Source data

We characterized the serum neutralizing activity of each group of immunized mice using authentic SARS-CoV-2 strains (Fig. 1k–p and Extended Data Fig. 1). As expected, serum from ChAd-Control-immunized mice did not neutralize any SARS-CoV-2 strain. Although monovalent ChAd-SARS-CoV-2-S induced robust neutralizing responses against WA1/2020 D614G (Fig. 1k), it elicited poor activity against Omicron variants (Fig. 1l–p). Whereas serum from ChAd-SARS-CoV-2-BA.5-S-immunized mice had relatively modest neutralizing responses against WA1/2020 D614G (Fig. 1k), greater activity was measured against BA.5 and BF.7 (Fig. 1l,m). However, the neutralization titers of BQ.1.1, XBB.1.1 and XBB.1.5 were reduced 5- to 32-fold (Fig. 1n–p). Serum from mice immunized with the bivalent vaccine had high neutralization titers against WA1/2020 D614G, BA.5 and BF.7, intermediate titers against BQ.1.1 and lower titers against XBB.1.1 and XBB.1.5. Neutralizing activity induced by the ChAd vaccines was also apparent following pairwise analysis of individual serum samples against all respective variants (Extended Data Fig. 2). Overall, serum from mice immunized with the bivalent vaccine broadly neutralized historical and antigenically distant SARS-CoV-2 strains, although inhibitory activity was more limited against the more recent Omicron lineage variants.

### ChAd vaccines induce mucosal IgG and IgA

**Fig. 2:**
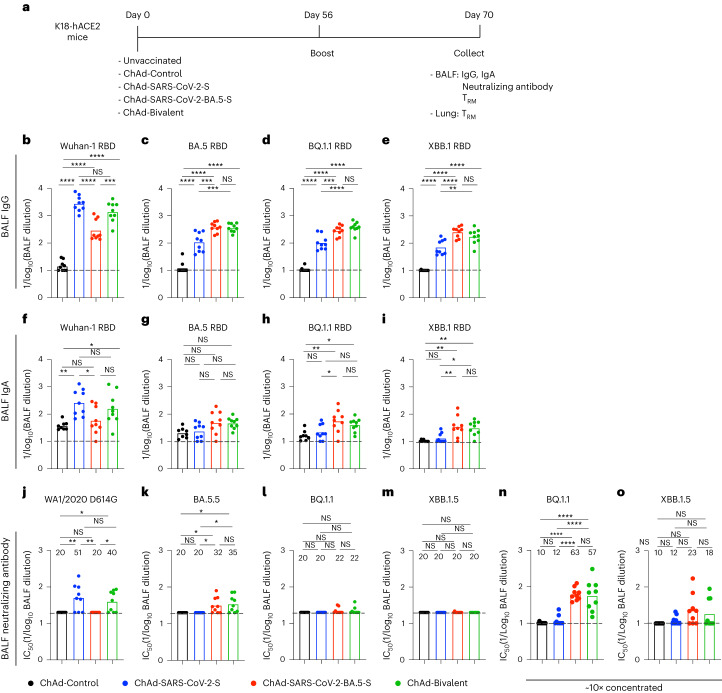
BALF antibody responses in K18-hACE2 mice after ChAd-vectored vaccine immunization. **a**, Scheme of experiments. **b**–**o**, Cohorts of 7- to 9-week-old female K18-hACE2 mice were immunized i.n. with ChAd-Control, ChAd-SARS-CoV-2-S, ChAd-SARS-CoV-2-BA.5-S or bivalent ChAd vaccine. Two weeks after boosting, BALF was collected, and antibody responses were evaluated. **b**–**i**, Titers of BALF RBD-specific IgG (**b**–**e**; *n* = 9, two experiments) and IgA (**f**–**i**; *n* = 8, 9, 9 and 9, left to right, two experiments). **j**–**m**, Neutralizing antibody titers of BALF against the indicated SARS-CoV-2 strains (*n* = 9, two experiments). **n**,**o**, Neutralizing antibody titers of concentrated (~10×) BALF against BQ.1.1 (**n**) and XBB.1.5 (**o**; *n* = 9, two experiments). Boxes illustrate geometric mean titers, and dotted lines show the LOD. Data were analyzed by one-way ANOVA with a Tukey’s post hoc test (**b-o)**; *P < 0.05, **P < 0.01, ***P < 0.001 and ****P < 0.0001; IC_50_, half-maximal inhibitory concentration. Source data

To assess for development of mucosal immunity in the respiratory tract, cohorts of 7-week-old female K18-hACE2 mice were immunized i.n. with ChAd-Control, ChAd-SARS-CoV-2-S, ChAd-SARS-CoV-2-BA.5-S or bivalent vaccine and boosted homologously, as we did previously^15^. Two weeks later, bronchoalveolar lavage fluid (BALF) was collected, and IgG and IgA responses against Wuhan-1, BA.5, BQ.1.1 and XBB.1 RBD proteins were measured (Fig. 2a). We observed a similar pattern of vaccine-induced IgG and IgA responses in BALF compared to serum (Fig. 2b–i): (1) mice immunized with ChAd-SARS-CoV-2-S had higher IgG and IgA titers against Wuhan-1 RBD than animals immunized with ChAd-SARS-CoV-2-BA.5-S, (2) ChAd-SARS-CoV-2-S elicited five- to tenfold lower IgG and IgA titers against the RBD of BA.5, BQ.1.1 or XBB.1 than against Wuhan-1, (3) ChAd-SARS-CoV-2-BA.5-S elicited higher IgG and IgA titers against the RBD of BA.5, BQ1.1 and XBB.1.5 S than ChAd-SARS-CoV-2-S, and (4) the bivalent vaccine induced more balanced IgG and IgA responses against all RBD proteins tested.

We also measured neutralizing antibody activity in BALF against WA1/2020 D614G, BA.5.5, BQ.1.1 and XBB.1.5 (Fig. 2j–m). Whereas ChAd-SARS-CoV-2-S induced low levels of neutralizing antibody against WA1/2020 D614G, the levels against all Omicron variants fell below the limit of detection (LOD) of the assay. Reciprocally, BALF from ChAd-SARS-CoV-2-BA.5-S-immunized mice had inhibitory activity against BA.5.5 but not WA1/2020 D614G or other Omicron variants. In BALF of mice immunized with the bivalent vaccine, low levels of neutralizing antibody were detected targeting WA1/2020 D614G and BA.5.5 but not BQ.1.1 or XBB.1.5. However, if BALF was concentrated tenfold, neutralizing activity against BQ.1.1 or XBB.1.5 was detected in more samples from mice immunized with vaccines containing ChAd-SARS-CoV-2-BA.5-S (Fig. 2n–o).

### ChAd vaccines induce T cell immunity in the respiratory tract

**Fig. 3:**
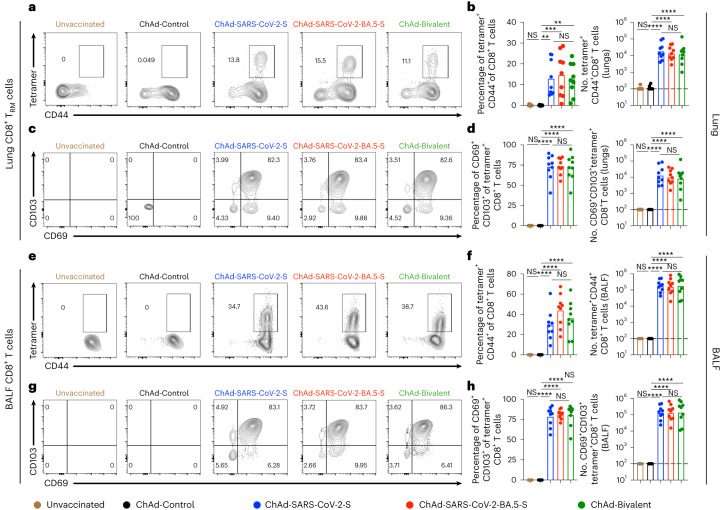
Mucosal memory T cell responses after ChAd vaccine immunization. K18-hACE2 mice were immunized i.n. and boosted as shown in Fig. 2a. **a**–**h**, Lung tissues (**a**–**d**) and BALF (**e**–**h**) were collected for T cell analysis by flow cytometry. Quantification of S-specific tetramer^+^CD8^+^ T cells (**a**,**b**) and CD69^+^CD103^+^tetramer^+^CD8^+^ T_RM_ cells (**c**,**d**) in lung tissues is shown as well as quantification of S protein-specific tetramer^+^CD8^+^ T cells (**e**,**f**) and CD69^+^CD103^+^tetramer^+^CD8^+^ T_RM_ cells (**g**,**h**) in BALF. Data in **a**, **c**, **e** and **g** are representative flow cytometry scatter plots, and data in **b** (*n* = 8, 9, 9, 9 and 9, left to right), **d** (*n* = 8, 9, 9, 9 and 9, left to right)**, f** (*n* = 8, 9, 9, 9 and 9, left to right) and **h** (*n* = 9, 9, 9, 9 and 9, left to right) show frequencies (left) and total cell numbers (right). Data are from two experiments. Boxes illustrate mean (frequencies) or geometric mean (total cell numbers) values, and dotted lines show the LOD. Data were analyzed by one-way ANOVA with a Tukey’s post hoc test; **P < 0.01, ***P < 0.001, ****P < 0.0001. Source data

**Extended Data Fig. 3:**
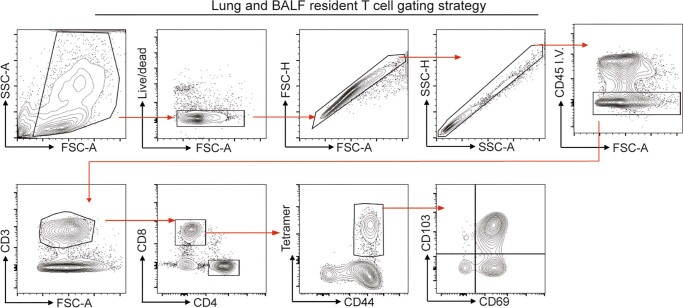
Flow cytometry gating strategies. Gating strategies to identify spike protein specific CD8^+^ T cells and resident memory CD8^+^ T cells in the lung and BALF. Single cell suspensions were gated for lymphocytes (forward scatter [FSC-A]/side scatter [SSC-A]), live cells (Viability dye eF506^−^), singlets (FSC-H/FSC-A), non-circulating T cells (IV-CD45^−^CD3^+^), CD8^+^T cells (CD4^−^ CD8^+^), followed by spike-specific CD8^+^ T cells (tetramer^+^CD44^+^) and resident memory CD8^+^ T cells (CD69^+^CD103^+^).

Memory T cells are hypothesized to contribute to protection against SARS-CoV-2 infection and disease, especially in the setting of poor neutralizing antibody responses^24–27^. CD69^+^CD103^+^ tissue-resident memory T (T_RM_) cells are local effector cells that rapidly respond to control respiratory viral infections^28–31^. To assess T_RM_ cell responses after i.n. immunization with ChAd-vectored vaccines (Fig. 2a), we collected BALF and lungs and analyzed cells by flow cytometry (Extended Data Fig. 3). Before tissue collection, we intravenously (i.v.) administered a fluorescently conjugated antibody to CD45 to label circulating cells and differentiate those from resident immune cells (i.v.-CD45^−^) in lung tissue. To identify S protein-specific CD8^+^ T cells, we stained cells with a major histocompatibility complex class I (MHC class I) tetramer that displays a conserved immunodominant peptide (S_539–546_; VNFNFNGL) in Wuhan-1, BA.5, BQ.1.1 and XBB.1.5 S proteins^18,32^. Whereas tetramer^+^CD8^+^ T cells were not detected in the lungs or BALF of unvaccinated or ChAd-Control-vaccinated mice, mice immunized with monovalent or bivalent ChAd vaccines showed high frequencies and numbers of (i.v.-CD45^−^) CD8^+^tetramer^+^ cells in the lung (Fig. 3a,b) and BALF (Fig. 3e,f). ChAd vaccines delivered i.n. induced tetramer^+^CD69^+^CD103^+^CD8^+^ T_RM_ cells in the lung (Fig. 3c,d) and similar cells in BALF (Fig. 3g,h) at comparable levels. Differences in the frequencies and numbers of peptide-specific lung-resident CD8^+^ T_RM_ cells were not observed after immunization with monovalent or bivalent ChAd vaccines.

### Immunization protects against SARS-CoV-2 infection in K18-hACE2 mice

**Fig. 4:**
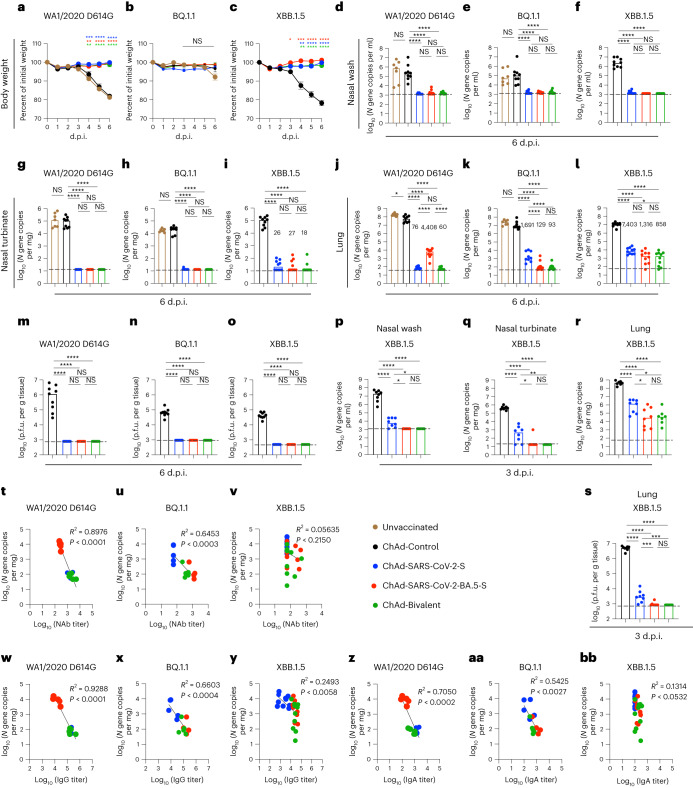
ChAd vaccines protect mice against infection by ancestral and Omicron SARS-CoV-2 strains. **a**–**o**, Seven- to 9-week-old female K18-hACE2 mice were immunized i.n. with 2 × 10^9^ viral particles of the indicated ChAd vaccines, as described in Fig. 1b. Eight weeks later, mice were challenged i.n. with 10^4^ f.f.u. of SARS-CoV-2 WA1/2020 D614G, BQ.1.1 or XBB.1.5 (two experiments). **a**–**c**, Body weight was measured over time and the percent of the initial weight was determined (two experiments). Data are shown as mean ± s.e.m. (WA1/2020 D614G, *n* = 9 mice per group; BQ.1.1, *n* = 9, 9, 10 and 10; XBB.1.5, *n* = 9, 10, 9 and 10 (left to right: ChAd-Control, ChAd-SARS-CoV-2-S, ChAD-SARS-CoV-2-BA.5-S and bivalent ChAd)). **d**–**l**, Viral RNA levels were determined at 6 d.p.i. in nasal washes (**d**–**f**), nasal turbinates (**g**–**i**) and lungs (**j**–**l**; WA1/2020 D614G: *n* = 7, 9, 8, 9 and 9; BQ.1.1: *n* = 7, 9, 9, 10 and 10; XBB.1.5: *n* = 9, 10, 9 and 10 (all left to right)). **m**–**o**, Infectious virus levels in the lungs (WA1/2020 D614G: *n* = 9, 8, 9 and 9; BQ.1.1: *n* = 9, 9, 10 and 10; XBB.1.5: *n* = 9, 10, 10 and 10 (all left to right)). Boxes illustrate median values, and dotted lines indicate the LOD. **p**–**s**, Viral burden at 3 d.p.i. after XBB.1.5 challenge (*n* = 8, 8, 8 and 7 mice per group (left to right), two experiments). Viral RNA levels in nasal washes (**p**), nasal turbinates (**q**) and lungs (**r**) are shown as well as infectious virus levels in the lungs (**s**). **t**–**bb**, Correlation analyses are shown comparing lung viral RNA levels to serum neutralizing antibody (NAb) titers (**t**–**v**), IgG titers (**w**–**y**) and IgA titers (**z**–**bb**). Data were analyzed by two-way ANOVA with a Tukey’s post hoc test (**a**–**c**), one-way ANOVA with a Tukey’s post hoc test (**d**–**s**) or linear regression analysis, with *P* and *R*^2^ values indicated (**t**–**bb**); *P < 0.05, **P < 0.01, ***P < 0.001 and ****P < 0.0001. Source data

We next performed challenge studies in K18-hACE2 mice, which are susceptible to most SARS-CoV-2 strains^33–36^. Cohorts of mice were immunized i.n. with a single dose of 2 × 10^9^ virus particles of ChAd-Control, ChAd-SARS-CoV-2-S, ChAd-SARS-CoV-2-BA.5-S or bivalent vaccine. Eight weeks later, mice were challenged i.n. with 10^4^ focus-forming units (f.f.u.) of WA1/2020 D614G, BQ.1.1 or XBB.1.5. Whereas substantial (20 to 25%) body weight loss was observed within 6 d of infection with WA1/2020 D614G or XBB.1.5 viruses in unvaccinated or ChAd-Control-immunized mice, weight loss was not seen after infection with BQ.1.1 (Fig. 4a–c); this result is consistent with experiments showing that some Omicron strains do not cause clinical disease in rodents^36–38^. All monovalent and bivalent ChAd-SARS-CoV-2 vaccines prevented weight loss caused by WA1/2020 D614G or XBB.1.5 infection.

In unvaccinated or ChAd-Control-vaccinated mice challenged with WA1/2020 D614G, BQ.1.1 or XBB.1.5, moderate to high amounts of viral RNA were measured at 6 d postinfection (d.p.i.) in nasal washes (Fig. 4d–f), nasal turbinates (Fig. 4g–i) and lungs (Fig. 4j–l). The monovalent or bivalent vaccines conferred robust protection against WA1/2020 D614G, BQ.1.1 or XBB.1.5 infection in the upper respiratory tract, with 10^2^- to 10^4^-fold reductions in viral RNA levels. Monovalent or bivalent vaccines also conferred robust protection against WA1/2020 D614G, BQ.1.1 or XBB.1.5 infection in the lungs with 10^3^- to 10^5^-fold reductions in viral RNA levels (Fig. 4j–l) and 10^2^- to 10^3^-fold reductions in infectious virus (Fig. 4m–o). Further analysis of viral RNA levels in the lungs revealed the following: (1) the ChAd-SARS-CoV-2-S vaccine conferred optimal protection against homologous WA1/2020 D614G infection but showed breakthrough after BQ.1.1 or XBB.1.5 challenge, (2) the ChAd-SARS-CoV-2-BA.5-S vaccine displayed optimal protection against BQ.1.1 and showed higher levels of viral RNA after WA1/2020 D614G or XBB.1.5 challenge, and (3) the ChAd-Bivalent vaccine showed broader protection against all challenge viruses, with lower levels of XBB.1.5 infection.

To further gauge the impact of ChAd-vectored vaccines, we performed viral burden analysis at 3 d.p.i., an earlier stage of XBB.1.5 infection before the appearance of clinical disease. ChAd-Control-vaccinated mice showed 10- to 100-fold higher amounts of viral RNA and infectious virus in nasal washes, nasal turbinates and lungs at 3 d.p.i. than at 6 d.p.i. (Fig. 4f,i,l,p–r). Mice immunized with the monovalent ChAd-SARS-CoV-2-S vaccine had lower levels (10^3^- to 10^4^-fold) of viral RNA in the upper and lower respiratory tracts (Fig. 4p–r) and lower levels of infectious virus in the lungs (Fig. 4s), although XBB.1.5 breakthrough infection was detected. The monovalent ChAd-SARS-CoV-2-BA.5-S and bivalent vaccines conferred greater protection against XBB.1.5 with virtually no viral RNA present in nasal washes and nasal turbinates and an approximately 26-fold reduction in viral RNA and no infectious virus in the lung (Fig. 4p–s).

We next examined whether there were correlations between levels of vaccine-induced antibody and viral load in the lung. Serum titers of neutralizing antibodies were inversely correlated with the amount of viral RNA in the lungs at 6 d.p.i. after challenge with WA1/2020 D614G or BQ.1.1 (Fig. 4t–u). The majority of WA1/2020 D614G breakthrough infections occurred in mice immunized with the ChAd-SARS-CoV-2-BA.5-S vaccine, which had lower serum neutralizing antibody titers against this virus. Reciprocally, most BQ.1.1 breakthrough infections occurred in mice immunized with ChAd-SARS-CoV-2-S that had low inhibitory titers in serum against BQ.1.1. By contrast, we failed to observe a correlation with XBB.1.5, as breakthrough infections in the lung were not linked to neutralizing antibody titers (Fig. 4v). We observed similar patterns when comparing serum titers of RBD IgG (Fig. 4w–y) or IgA (Fig. 4z–bb) to the amount of viral RNA in the lung. Thus, although serum antibody responses predict the protective activity of monovalent and bivalent ChAd vaccines for WA1/2020 D614G and BQ.1.1 challenge, they did not for XBB.1.5 infection.

**Fig. 5:**
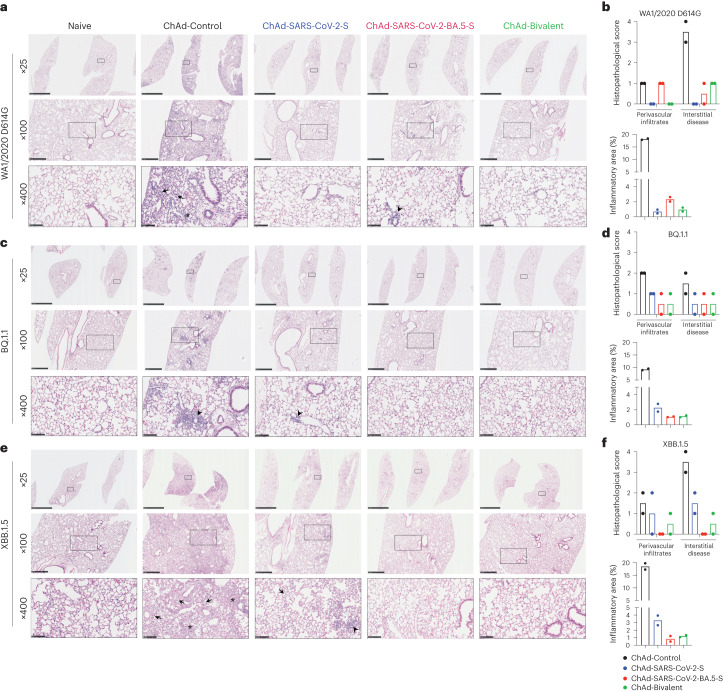
ChAd vaccines protect mice against SARS-CoV-2-induced lung pathology. Seven- to 9-week-old female K18-hACE2 mice were immunized with ChAd vaccines and challenged with WA1/2020 D614G, BQ.1.1 or XBB.1.5, as described in Fig. 1b. Hematoxylin and eosin staining of lung sections (**a**, **c** and **e**) and corresponding pathology scores (**b**, **d** and **f**) using two different analysis modes (see Methods) are shown. Data for WA1/2020 D614G (**a** and **b**), BQ.1.1 (**c** and **d**) and XBB.1.5 (**e** and **f**) are shown. Images show low-power (×25; scale bars, 2.5 mm), medium-power (×100; scale bars, 500 µm) and high-power (×400; scale bars, 100 µm) magnifications. Images at high magnifications (×400) show infected lungs with immune cell infiltrates (arrows), thickened septa and consolidated air space (asterisks) and foci of immune cells near perivasular and peribronchial spaces (arrow heads). Images are representative of *n* = 2 mice per group. Source data

ChAd vaccines protect against lung injury in K18-hACE2 mice. After WA1/2020 D614G or XBB.1.5 challenge, ChAd-Control-immunized mice developed interstitial pneumonia with extensive immune cell infiltration, alveolar space consolidation, vascular congestion and edema (Fig. 5a,b,e,f). By comparison, and consistent with decreased virulence of some Omicron strains in mice^36–38^, after BQ.1.1 challenge, lungs from ChAd-Control-immunized mice showed patchy focal immune cell infiltration and air space consolidation principally in perivascular and peribronchial regions (Fig. 5c,d). Mice immunized with either monovalent or bivalent ChAd-SARS-CoV-2 vaccines showed virtually complete protection against lung pathology after WA1/2020 D614G or BQ.1.1 challenge, with few histological changes (Fig. 5a–d). By contrast, whereas lung sections from mice immunized with ChAd-SARS-CoV-2-BA.5-S or bivalent vaccine appeared normal following XBB.1.5 challenge, those from ChAd-SARS-CoV-2-S-immunized mice showed foci of immune cell infiltration in the periphery of the lung (Fig. 5e, third column). Thus, while i.n. immunization with monovalent or bivalent ChAd vaccines generally conferred robust protection against SARS-CoV-2-induced lung pathology, BA.5-targeted vaccines performed better against the antigenically distant XBB.1.5 strain.

**Extended Data Fig. 4:**
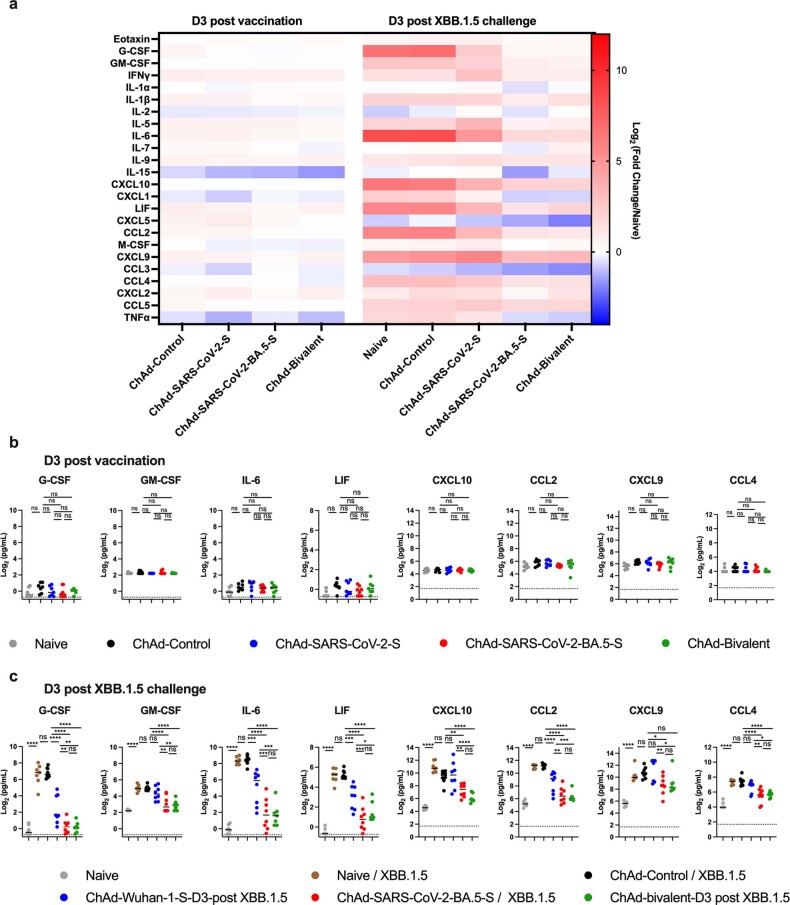
Cytokine and chemokine responses in the lungs of ChAd vaccinated and XBB.1.5-infected mice. Seven to nine-week-old female K18-hACE2 mice were immunized IN with ChAd vaccines and challenged IN with 10^4^ f.f.u. of XBB.1.5. (**a**) Heat maps of cytokine and chemokine levels in lung homogenates at 3 days after vaccination or 3 days after XBB.1.5 infection. Fold-change was calculated relative to naive uninfected mice, and log_2_ fold differences are presented. (**b-c**) Cytokine and chemokine concentrations (pg/mL) in lung homogenates 3 days after vaccination only (**b**, n = 7, 7, 7, 8, 7 (left-to-right)) and 3 days after challenge with XBB.1.5 (**c**, n = 7, 8, 8, 8, 7 (left-to-right)). Individual data points are from two experiments. Data also are shown in Supplementary Table 1. Statistical analysis: one-way ANOVA with a Tukey’s post hoc test (**b-c**): * *P* < 0.05, **P < 0.01, ***P < 0.001, ****P < 0.0001. Source data

**Extended Data Fig. 5:**
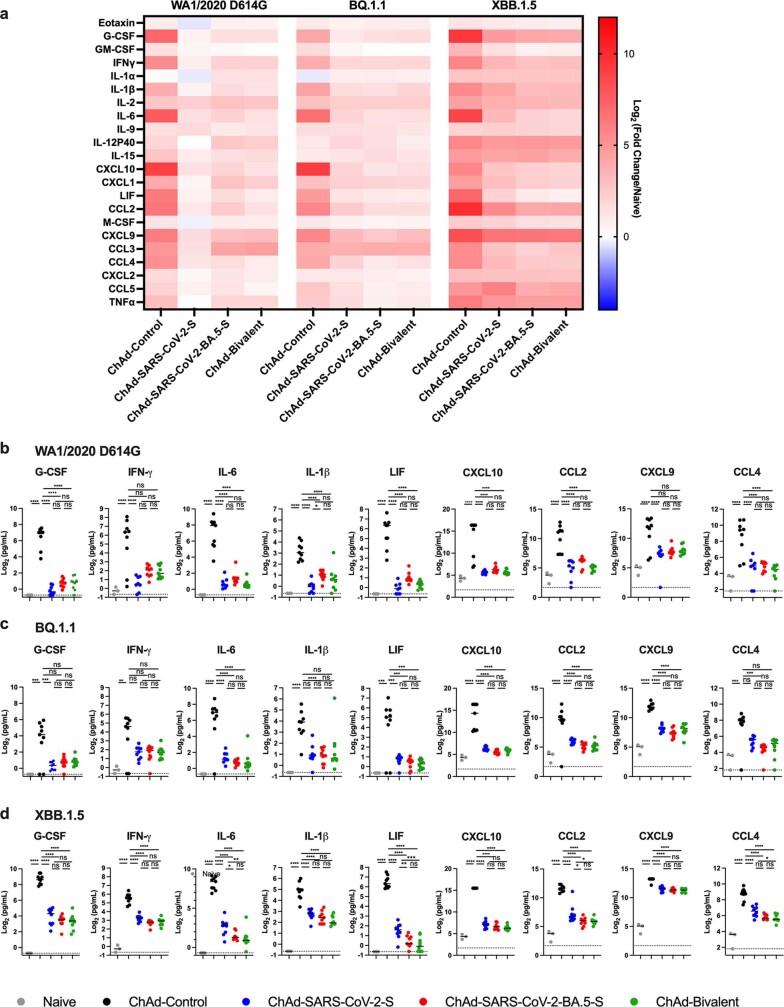
Cytokine and chemokine responses in the lungs of ChAd vaccinated and WA1/2020 D614G, BQ.1.1 or XBB.1.5-infected mice. Seven to nine-week-old female K18-hACE2 mice were immunized IN with ChAd vaccines and challenged IN with WA1/2020 D614G, BQ.1.1 or XBB.1.5 as described in Fig. 1b. Cytokine and chemokine levels in lung homogenates at 6 dpi were determined using a multiplexed platform. (**a**) Heat maps of cytokine and chemokine levels. Fold-change was calculated relative to naive uninfected mice, and log2 fold differences are presented. (**b-d**) Cytokine and chemokine concentrations (pg/mL) in lung homogenates after challenge with WA1/2020 D614G (**b**, n = 3, 9, 8, 9, 9 (left-to-right)), BQ.1.1 (**c**, n = 3, 9, 9, 10, 10 (left-to-right)), or XBB.1.5 (**d**, n = 3, 9, 10, 9, 10 (left-to-right)). Individual data points are from two experiments. Data also are shown in Supplementary Table 2. Statistical analysis: one-way ANOVA with a Tukey’s post hoc test (b-d): * P < 0.05, ∗∗ P < 0.01, ∗∗∗ P < 0.001, ∗∗∗∗ P < 0.0001. Source data

Because a hyperinflammatory response contributes to severe COVID-19 and lung disease^39,40^, we measured cytokines and chemokines in lung homogenates after ChAd immunization and virus infection. At 3 d after vaccination, the levels of cytokines and chemokines in the lungs of ChAd vector-immunized animals were virtually the same as in naive mice (Extended Data Fig. 4a,b), indicating that the ChAd vectors alone do not induce lower airway inflammation. By contrast, at 3 d after XBB.1.5 challenge, proinflammatory responses were elicited in the lungs of unvaccinated or ChAd-Control-vaccinated mice (Extended Data Fig. 4a,c). Lower levels of cytokines and chemokines were detected in mice vaccinated with ChAd-SARS-CoV-2-S vaccines. Monovalent ChAd-SARS-CoV-2-BA.5-S and bivalent vaccines conferred superior protection against XBB.1.5-induced inflammation compared to monovalent ChAd-SARS-CoV-2-S vaccine, with lower levels of several analytes (Extended Data Fig. 4a,c). We also assessed inflammatory responses at 6 d.p.i. with the full panel of WA1/2020 D614G, BQ.1.1 and XBB.1.5 viruses. Whereas expression of several cytokines and chemokines remained elevated in lung homogenates of ChAd-Control-immunized mice, levels were decreased in mice vaccinated with either monovalent or bivalent ChAd vaccines, with relatively small differences observed among groups regardless of the challenge virus (Extended Data Fig. 5a–d). Thus, i.n. immunization with monovalent or bivalent ChAd-vectored vaccines alone elicits negligible cytokine responses but protects against severe lung inflammation induced by ancestral or Omicron strains of SARS-CoV-2 in mice.

**Fig. 6:**
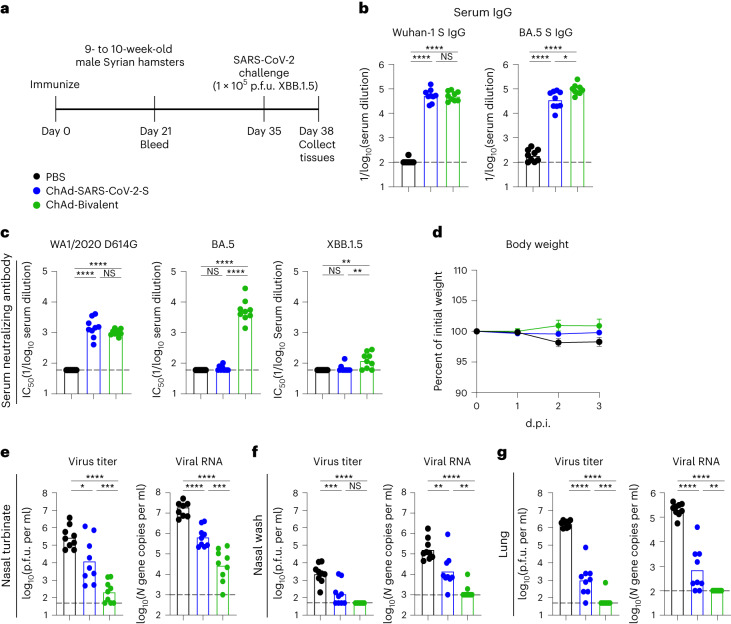
ChAd vaccines protect Syrian hamsters against XBB.1.5 infection. **a**, Experimental design. **b**, Anti-Wuhan-1 and anti-BA.5 S protein responses in the sera of hamsters immunized i.n. with monovalent ChAd-SARS-CoV-2-S (blue) or bivalent ChAd vaccine (green). Animals immunized with PBS were used as controls (black). Serum was collected 21 d after immunization. **c**, Serum neutralizing antibody responses against WA1/2020 D614G, BA.5 and XBB1.5. **d**, Weight loss/gain (percent of initial weight) (mean ± s.e.m.) of hamsters challenged with SARS-CoV-2. **e**–**g**, Infectious virus titers and viral RNA levels determined 3 d after XBB.1.5 challenge in nasal turbinates (**e**), nasal washes (**f**) and lungs (**g**). Bars indicate the geometric mean values, and dotted lines denote the LOD of the assays (two experiments, *n* = 9 for all assessments). Data in **b**, **c** and **e**–**g** were analyzed by one-way ANOVA with a Tukey’s post hoc test; *P < 0.05, **P < 0.01, ***P < 0.001 and ****P < 0.0001. Source data

ChAd vaccines protect against XBB.1.5 infection in hamsters. We next evaluated the protective efficacy of our ChAd vaccines against challenge with the XBB.1.5 variant in Syrian hamsters. Groups of 9- to 10-week-old male hamsters were immunized once i.n. with 10^10^ virus particles of monovalent ChAd-SARS-CoV-2-S or bivalent ChAd vaccine (Fig. 6a). Aged-matched hamsters that received PBS immunizations served as controls. Serum was collected 21 d later, and Wuhan-1 and BA.5 S-specific IgG antibodies were measured (Fig. 6b). Hamsters immunized with monovalent ChAd-SARS-CoV-2-S had high serum IgG titers against Wuhan-1 and BA.5 S proteins. Immunization with bivalent ChAd vaccine induced similar levels of Wuhan-1 S-specific IgG antibodies but higher IgG responses to BA.5 S protein than the monovalent vaccine (2.7-fold). We also characterized the neutralizing activity of serum from ChAd-immunized hamsters (Fig. 6c). Whereas ChAd-SARS-CoV-2-S induced robust neutralizing responses against WA1/2020 D614G, the titers against both Omicron variants fell below the LOD of the assay. Serum from hamsters immunized with the bivalent vaccine had high neutralizing titers against WA1/2020 D614G and BA.5 yet much lower titers against XBB.1.5.

Five weeks after immunization, the hamsters were challenged i.n. with 10^5^ plaque-forming units (p.f.u.) of XBB.1.5, and animal weight was measured for 3 d. Unvaccinated hamsters inoculated with XBB.1.5 lost only a small amount (~3%) of weight. Immunization with monovalent ChAd-SARS-CoV-2-S or bivalent ChAd vaccines protected against weight loss after XBB.1.5 infection, but the differences did not reach statistical significance (Fig. 6d). We next quantified the impact of vaccination on viral infection. Compared to control animals, we detected 22-fold and 1,300-fold lower infectious virus titers and 29-fold and 700-fold lower viral RNA levels in the nasal turbinates of hamsters immunized with monovalent and bivalent vaccines, respectively (Fig. 6e). In nasal washes, we detected 14-fold and 47-fold lower infectious virus titers and 11-fold and 118-fold lower viral RNA levels in hamsters immunized with monovalent and bivalent vaccines, respectively, than in control animals (Fig. 6f). A similarly improved virological outcome was seen in the lungs of XBB.1.5-challenged hamsters after immunization with ChAd vectors encoding S proteins (Fig. 6g). Compared to control animals, immunization with monovalent or bivalent ChAd reduced infectious viral titers 1,600-fold and 25,000-fold, respectively, and viral RNA levels 290-fold and 2,100-fold, respectively. Consistently, the bivalent ChAd vaccine showed better inhibitory activity against XBB.1.5 infection in the upper and lower respiratory tracts of hamsters than the monovalent vaccine encoding Wuhan-1 S protein.

### Vaccine-induced serum antibody does not protect against the XBB.1.5 strain

**Fig. 7:**
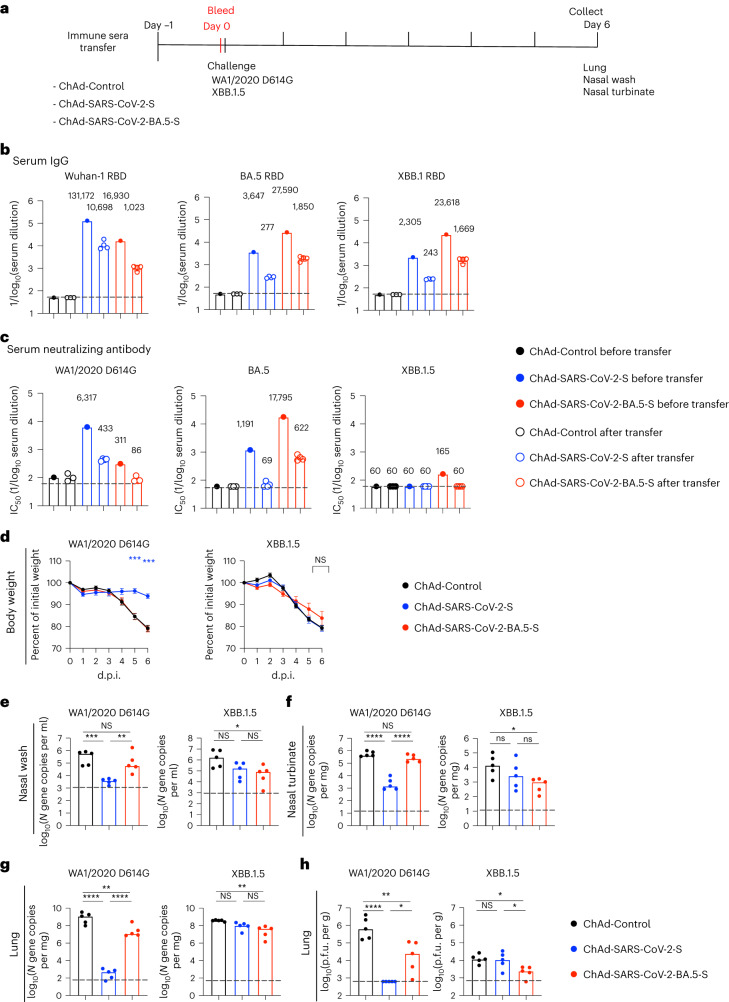
Vaccine-induced serum antibody responses are insufficient to protect against XBB.1.5. **a**, Scheme of serum transfer, blood collection and virus challenge. Seven- to 8-week-old female K18-hACE2 mice were administered pooled sera collected from mice immunized with ChAd-Control, ChAd-SARS-CoV-2-S or ChAd-SARS-CoV-2-BA.5-S vaccine (as described in Fig. 1b) by i.p. injection. **b**,**c**, Blood was collected from a subset of animals 24 h later, and serum antibody levels were determined. **b**, RBD-specific IgG to Wuhan-1, BA.5 and XBB.1. **c**, Serum neutralizing antibody titers against the indicated authentic SARS-CoV-2 strains (ChAd-Control, *n* = 3; ChAd-SARS-CoV-2-S, *n* = 4; ChAd-SARS-CoV-2-BA.5-S, *n* = 4). Boxes illustrate geometric mean values, numbers on top of the bar graph represent geometric mean values, and dotted lines show the LOD. **d**–**h**, One day after serum transfer, mice were challenged with 10^4^ f.f.u. of WA1/2020 D614G or XBB.1.5 i.n. **d**, Body weight measurements (percent of initial weight) (mean ± s.e.m.; *n* = 5 mice per group). **e**–**g**, Levels of viral RNA in nasal washes (**e**), nasal turbinates (**f**) and lungs (**g**). **h**, Infectious virus in the lungs at 6 d.p.i. were analyzed (*n* = 5 mice per group). Boxes indicate median values, and dotted lines show the LOD. Data were analyzed by two-way ANOVA with a Tukey’s post hoc test (**d**) or one-way ANOVA with a Tukey’s post hoc test (**e**–**h**); *P < 0.05, **P < 0.01, ***P < 0.001 and ****P < 0.0001. Source data

To begin to define the dominant mechanism of ChAd vaccine-mediated protection against heterologous XBB.1.5 challenge, we tested whether cross-reactive antibodies in circulation were sufficient to mediate protection. We passively transferred 100 μl of pooled serum from mice vaccinated with ChAd-Control, ChAd-SARS-CoV-2-S or ChAd-SARS-CoV-2-BA.5-S to naive recipient K18-hACE2 mice and 24 h later challenged them i.n. with 10^4^ f.f.u. of WA1/2020 D614G or XBB.1.5 (Fig. 7a). We first measured serum antibody levels in recipient mice 24 h after passive transfer. In general, IgG concentrations against the RBD of Wuhan-1, BA.5 or XBB.1 were approximately 10- to 20-fold lower than levels in the pooled serum before transfer due to a dilution effect (Fig. 7b). The neutralizing antibody titers in animals receiving pooled sera from ChAd-SARS-CoV-2-S-vaccinated mice were >1:400 against WA1/2020 D614G but were undetectable against BA.5 and XBB.1.5 (Fig. 7c). Analogously, the neutralizing antibody titers in animals receiving pooled sera from ChAd-SARS-CoV-2-BA.5-S-vaccinated mice were >1:600 against BA.5 but were undetectable against WA1/2020 D614G and XBB.1.5.

We next performed virus challenge experiments in recipient mice. As expected, substantial (20 to 25%) weight loss was observed in mice that received sera from ChAd-Control-immunized animals and were challenged with WA1/2020 D614G or XBB.1.5 (Fig. 7d). Although sera from ChAd-SARS-CoV-2-S-immunized animals protected K18-hACE2 mice from weight loss following homologous WA1/2020 D614G challenge, it did not protect against XBB.1.5 challenge (Fig. 7d). Sera from ChAd-SARS-CoV-2-BA.5-S-immunized animals failed to protect against weight loss after challenge with either WA1/2020 D614G or XBB.1.5 (Fig. 7d). In mice that received sera from ChAd-Control-immunized animals, high to moderate levels of WA1/2020 D614G or XBB.1.5 viral RNA were present in nasal washes, nasal turbinates and lungs at 6 d.p.i. (Fig. 7e–g). Sera from ChAd-SARS-CoV-2-S-immunized mice substantially reduced the levels of WA1/2020 D614G RNA in nasal washes, nasal turbinates and lungs (Fig. 7e–g) and infectious virus in the lung (Fig. 7h). However, XBB.1.5 RNA and infectious virus levels in the upper and lower respiratory tracts were not different between animals that received sera from ChAd-Control-immunized mice and those that received sera from ChAd-SARS-CoV-2-S-immunized mice (Fig. 7e–g). Passive transfer of sera from ChAd-SARS-CoV-2-BA.5-S-immunized mice also did not confer protection against heterologous WA1/2020 D614G infection in nasal washes and nasal turbinates, although reduced levels of viral RNA and infectious virus were observed in the lungs (Fig. 7e–h). Moreover, mice that received sera from ChAd-SARS-CoV-2-BA.5-S-immunized animals had limited protection against XBB.1.5 infection and showed only 20- to 30-fold reductions in viral RNA in nasal washes, nasal turbinates and lungs and 5-fold decreases in infectious virus in the lungs (Fig. 7e–h). Collectively, these results suggest that, although ChAd vaccine-elicited serum antibody responses are sufficient to confer protection against matched SARS-CoV-2 strains, additional immune mechanisms beyond serum antibody responses likely contribute to ChAd vaccine-mediated protection against the antigenically distant XBB.1.5 strain.

### Vaccine-induced CD8^+^ T cells control XBB.1.5 infection in the respiratory tract

**Extended Data Fig. 6:**
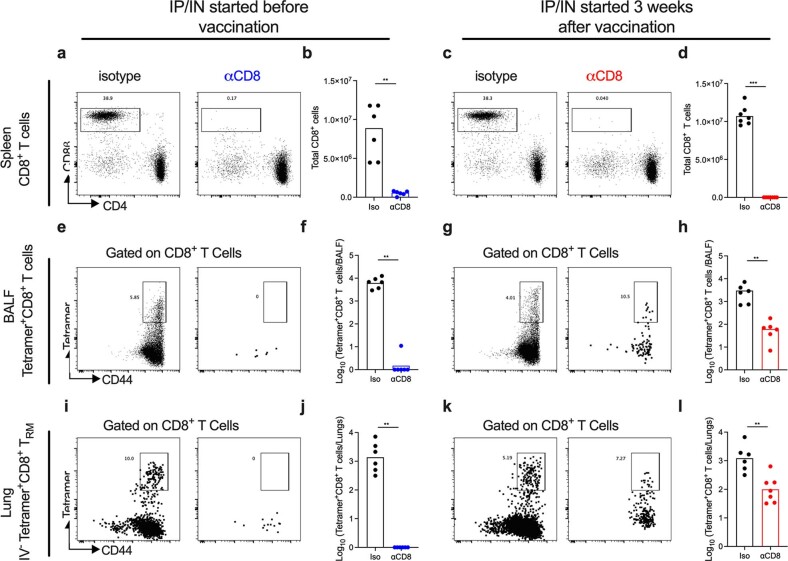
Depletion of CD8^+^ T cells in different tissue compartments. Seven-week-old female K18-hACE2 mice were immunized via IN route with a single dose of ChAd-SARS-CoV-2-S vaccine and treated with anti-CD8 or isotype antibodies via different regimens: IP administration beginning one day before vaccination and every 4 days thereafter plus IN administration beginning at D-1 and D6 after vaccination and also given at days 26 and 29 (**a-b, e-f, i-j**); or IP and IN administration given 26 and 29 days after vaccination (**c-d, g-h, k-l**). On day 31 after immunization, spleens (**a-d**), BALF (**e-h**) and lungs (**i-l**) were analyzed by flow cytometry for CD8^+^ T cell and spike-tetramer^+^ cell frequencies and numbers. Representative scatter plots are shown (**a, c, e, g, i, k**) as well as total cell numbers (**b, d, f, h, j, l**). Data are from two experiments (**b**, n = 6; **d**, n = 7; **f**, n = 6; **h**, n = 6; **j**, n = 6; **l**, n = 6, 7 (left-to-right); boxes illustrate geometric mean values). Statistical analysis: one-way ANOVA with Tukey’s post-test: * *P* < 0.05, ^**∗∗**^ *P* < 0.01, ^**∗∗∗**^ *P* < 0.001, ^**∗∗∗∗**^ *P* < 0.0001. Source data

We next evaluated the role of CD8^+^ T cells in vaccine-mediated protection against XBB.1.5 challenge. We first performed an experiment to assess our ability to deplete CD8^+^ T cells, in particular the respiratory tract-resident cells induced by nasal vaccination (Extended Data Fig. 6). K18-hACE2 mice were immunized i.n. with a single dose of ChAd-SARS-CoV-2-S vaccine and treated with anti-CD8 or isotype antibodies via two different regimens: (1) intraperitoneal (i.p.) administration beginning 1 d before vaccination and every 4 d thereafter plus i.n. administration 1 d before and 6 d after vaccination and also at days 26 and 29 (i.p./i.n., day –1 to day 30) or (2) i.p. and i.n. administration given 26 and 29 d after vaccination (i.p./i.n., day 26 and day 29). On day 31 after immunization, spleens, BALF and lungs (Extended Data Fig. 6a–l) were analyzed for CD8^+^ T cells by flow cytometry. Animals that received anti-CD8 starting before vaccination showed a nearly complete loss of CD8^+^ T cells in the spleen and SARS-CoV-2 tetramer^+^CD8^+^ T cells in the BALF and within the lung parenchyma (i.v.-CD45^–^ (T_RM_ cells); Extended Data Fig. 6i,j). Mice receiving anti-CD8 starting at day 26 showed nearly complete depletion of CD8^+^ T cells in the spleen and tetramer^+^CD8^+^ T cells in the BALF (98%; Extended Data Fig. 6g,h) and robust, yet less complete, depletion of tetramer^+^CD8^+^ T cells in the lung parenchyma (90% reduction; i.v.-CD45^–^; Extended Data Fig. 6k,l).

**Fig. 8:**
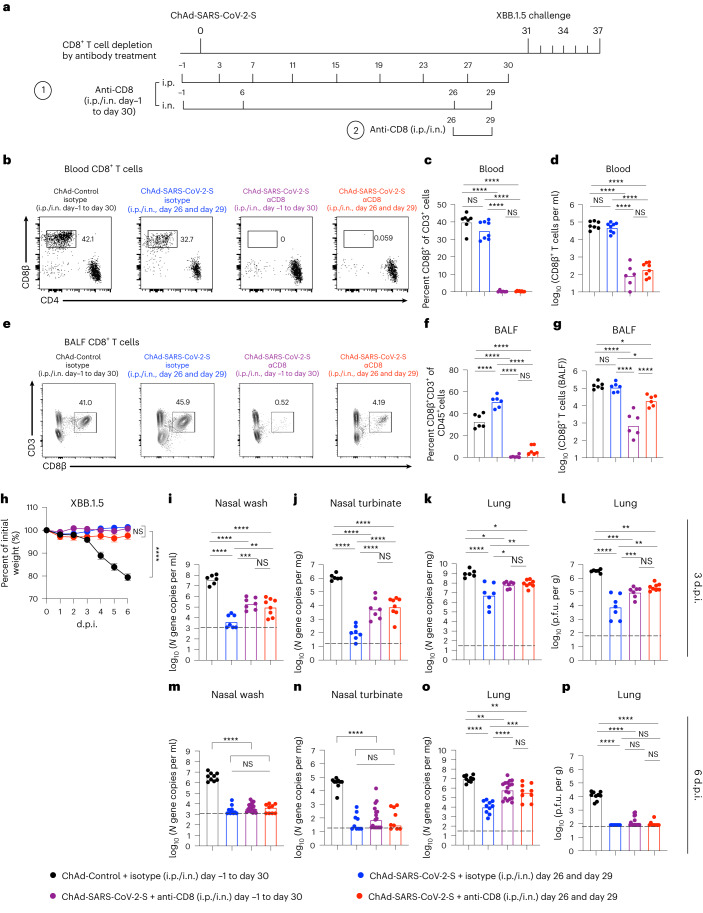
ChAd-SARS-CoV-2-S vaccine-induced CD8^+^ T cells control XBB.1.5 respiratory tract infection. **a**, Scheme of immunizations, CD8^+^ T cell depletion and XBB.1.5 challenge. Cohorts of 7-week-old female K18-hACE2 mice were immunized i.n. with a single dose (2 × 10^9^ viral particles) of ChAd-Control or ChAd-SARS-CoV-2-S vaccine and treated with CD8-depleting or isotype control antibody via i.p. plus i.n. administration (i.p./i.n.) as indicated. Thirty-one days after vaccination, mice were challenge with 10^4^ f.f.u. of XBB.1.5 i.n. **b**–**g**, Representative flow cytometry plots (**b** and **e**), frequencies (**c** and **f**) and numbers (**d** and **g**) of CD8^+^ T cells in the blood 1 d before (**c** and **d**) or in BALF 6 d after (**f** and **g**) XBB.1.5 challenge (**c** and **d**: *n* = 7, 6, 6 and 8 (left to right); **f** and **g**: *n* = 6 per group). **h**, Body weight measurements (percent of initial weight) (mean ± s.e.m.; ChAd-Control + isotype day –1 to day 30, *n* = 9; ChAd-SARS-CoV-2-S + isotype day 26 and day 29, *n* = 10; ChAd-SARS-CoV-2-S + anti-CD8 day –1 to day 30, *n* = 17; ChAd-SARS-CoV-2-S + anti-CD8 day 26 and day 29, *n* = 9). **i**–**p**, At 3 d.p.i. (**i**–**l**: *n* = 6, 7, 7 and 8 (left to right)) or 6 d.p.i. (**m**–**p**; *n* = 9, 9, 17 and 9 (left to right)), viral RNA levels in nasal washes (**i** and **m**), nasal turbinates (**j** and **n**) and lungs (**k** and **o**) and infectious virus levels in lungs (**l** and **p**) were measured. Boxes indicate median values, and dotted lines show the LOD. Data were analyzed by one-way ANOVA with a Tukey’s post hoc test (**c**, **d**, **f**, **g** and **i**–**p**) or two-way ANOVA with a Tukey’s post hoc test (**h**); *P < 0.05, **P < 0.01, ***P < 0.001 and ****P < 0.0001. Source data

**Extended Data Fig. 7:**
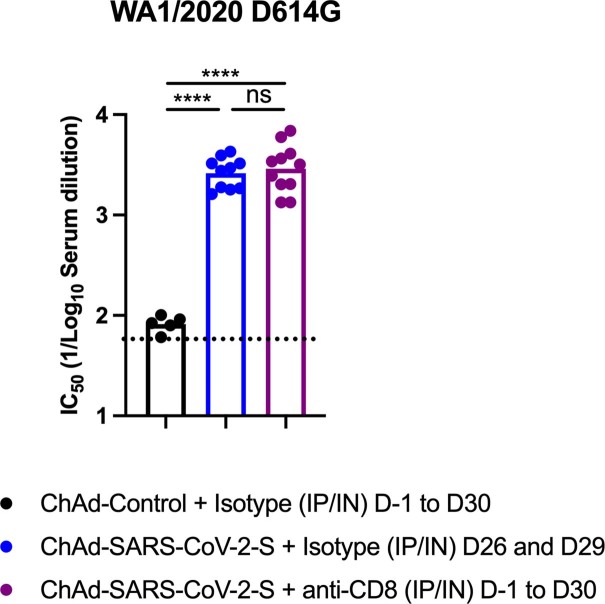
CD8^+^ T cell depletion does not impair vaccine-induced neutralizing antibody responses. Cohorts of 7-week-old female K18-hACE2 mice were immunized via IN route with a single dose (2 × 10^9^ vp) of ChAd-Control or ChAd-SARS-CoV-2-S vaccine and treated with anti-CD8 or isotype control antibody via IP and IN route as shown in Fig. 7f. Sera were collected four weeks after immunization, and neutralizing antibody responses were determined against SARS-CoV-2 WA1/2020 D614G (n = 5 for ChAd-Control; n = 10 for ChAd-SARS-CoV-2-S + isotype control antibody and ChAd-SARS-CoV-2-S + anti-CD8 antibody, two experiments, boxes illustrate geometric mean values, dotted lines show the LOD. Statistical analysis: one-way ANOVA with Tukey’s post-test: *P < 0.05, **P < 0.01, ***P < 0.001, ****P < 0.0001. Source data

Given these results, we evaluated the role of CD8^+^ T cells in protection against XBB.1.5 challenge after i.n. immunization. Cohorts of 7-week-old K18-hACE2 mice were immunized i.n. with a single dose of ChAd-Control or ChAd-SARS-CoV-2-S vaccine and depleted of CD8^+^ T cells (Fig. 8a). One day before XBB.1.5 challenge (day 30), we confirmed CD8^+^ T cell depletion in the blood of animals (Fig. 8b–d) and that this treatment did not affect serum neutralizing antibody responses (Extended Data Fig. 7). We also assessed CD8^+^ T cell responses in the BALF at 6 d after XBB.1.5 challenge. As expected, compared to the isotype control groups, the fraction and numbers of CD8^+^ T cells in the BALF were markedly lower in mice receiving anti-CD8 (Fig. 8e–g).

On day 31, all mice were challenged i.n. with 10^4^ f.f.u. of XBB.1.5. Although ChAd-Control-immunized mice that received isotype control antibody showed substantial weight loss, this was not observed in ChAd-SARS-CoV-2-S-immunized mice that received either isotype control or anti-CD8 (Fig. 8h). We next assessed the impact of vaccine-induced CD8^+^ T cells on viral infection at 3 (Fig. 8i–l) and 6 d.p.i. (Fig. 8m–p). In ChAd-Control-immunized mice that received isotype control antibody, high amounts of XBB.1.5 RNA were detected in nasal washes and nasal turbinates (Fig. 8i,j,m,n). In the upper respiratory tract of ChAd-SARS-CoV-2-S-vaccinated mice treated with isotype control antibody, viral RNA levels were markedly lower in nasal washes and nasal turbinates at 3 (10^4^- to 10^5^-fold) and 6 d.p.i. (10^3^- to 10^4^-fold). Depletion of CD8^+^ T cells either before or after vaccination resulted in approximately 100-fold higher viral RNA levels in nasal washes and nasal turbinates at 3 d.p.i. (Fig. 8i,j) but only marginal differences at 6 d.p.i. (Fig. 8m,n). In the lungs of ChAd-Control-vaccinated mice that received isotype control antibody, high amounts of XBB.1.5 RNA and infectious virus were measured at 3 (Fig. 8k,l) and 6 d.p.i. (Fig. 8o,p), and the viral burden was reduced by 100- to 1,000-fold at 3 and 6 d.p.i. in mice vaccinated with ChAd-SARS-CoV-2-S that received isotype control antibody (Fig. 8k,l,o,p). Depletion of CD8^+^ T cells in ChAd-SARS-CoV-2-S-immunized mice resulted in 15-fold higher levels of viral RNA and infectious virus in the lungs at 3 d.p.i. (Fig. 8k,l) and larger 35- to 62-fold increases at 6 d.p.i. (Fig. 8o), indicating that the anamnestic CD8^+^ T cell response contributes to viral control in the lung. In general, we did not observe substantive differences in viral burden between groups receiving CD8-depleting antibodies before or after vaccination.

**Extended Data Fig. 8:**
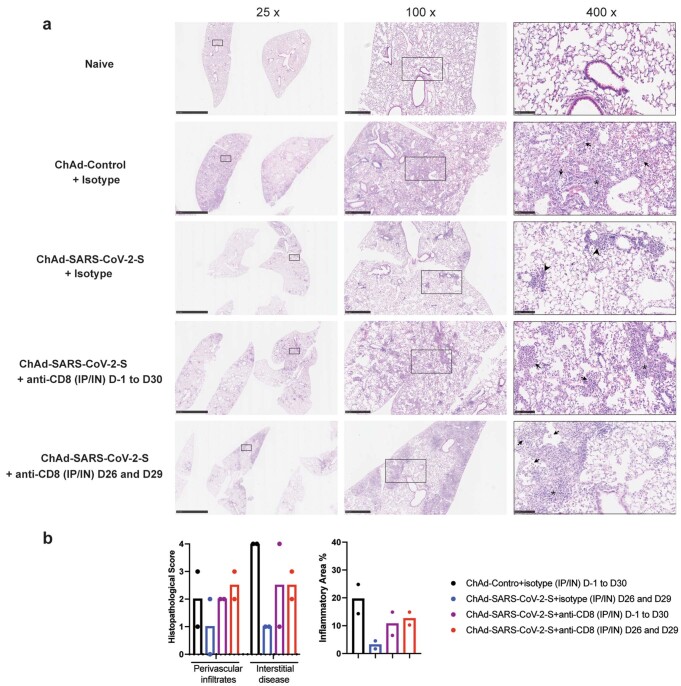
Lung pathology in ChAd vaccine-immunized K18-hACE2 mice after XBB.1.5 challenge and depletion of CD8^+^ T cells. K18-hACE2 mice were immunized, treated with anti-CD8 depletion or isotype control antibodies, and then challenged with XBB.1.5 as described in Fig. 8a. Hematoxylin and eosin staining of lung sections (**a**) and corresponding pathological scores using two different analysis modes (see Methods) are shown (**b**). Images show low- (scale bars, 2.5 mm), medium- (scale bars, 500 µm) and high-power (scale bars, 100 µm) magnification. Right panels at high magnifications (400x) show infected lungs with immune cell infiltrates (arrows), thickened septa and consolidated airspace (asterisks), and foci of immune cells around perivasular and peribronchial spaces (arrow heads). Representative images from n = 2 mice per group. Note, the sections from naïve animals are provided as a comparative reference and are from historical specimens shown in Fig. 5. Source data

Finally, we evaluated the impact of CD8^+^ T cell depletion on vaccine-mediated protection against virus-induced lung injury by performing histological analysis at 6 d.p.i. After XBB.1.5 challenge, lungs from ChAd-Control-immunized mice that received isotype control antibody showed evidence of interstitial pneumonia with extensive immune cell infiltration and alveolar space consolidation (Extended Data Fig. 8a). Mice immunized with ChAd-SARS-CoV-2-S vaccine that received isotype control antibody showed less inflammation with only small foci of immune cells principally in perivascular and peribronchial regions (Extended Data Fig. 8a). By contrast, mice immunized with ChAd-SARS-CoV-2-S that received anti-CD8 showed more lung injury and pneumonitis (Extended Data Fig. 8a). These results validate a role for CD8^+^ T cells in vaccine-mediated protection against heterologous XBB.1.5 infection.

## Discussion

We compared the immunogenicity and protective activity of i.n. delivered monovalent or bivalent ChAd-vectored vaccines against ancestral WA1/2020 D614G and two Omicron strains (BQ.1.1 and XBB.1.5) in K18-hACE2 transgenic mice and hamsters. The i.n. delivered ChAd-vectored SARS-CoV-2 vaccines induced robust systemic IgG and IgA responses in mice, with the monovalent vaccines generating better responses against viruses that matched the immunizing antigen. The bivalent vaccine induced more balanced serum IgG, IgA and neutralizing responses against ancestral and several Omicron strains in mice and hamsters. These results are consistent with studies showing that intramuscularly delivered bivalent SARS-CoV-2 mRNA vaccine boosters elicit antibody responses with greater breadth in humans and in animal models^41–46^ and confer greater protection than monovalent boosters targeting the ancestral strain. Bivalent ChAd vaccine delivered i.n. also induced broadly reactive IgG and IgA responses in the BALF of mice. Notwithstanding these data, antibody neutralizing responses in serum or BALF against XBB.1 strains were markedly lower, even with ChAd vaccines encoding the BA.5 S antigen, consistent with reports of humans receiving BA.5 bivalent boosters^11,12,47^. This is likely because the S proteins of XBB.1 lineage strains have additional mutations that escape neutralizing antibodies generated against Wuhan-1 or even BA.4/BA.5 S antigens. Despite inducing neutralizing antibodies that poorly cross-react with or inhibit XBB.1.5, the i.n. delivered ChAd vaccines conferred clinical and virological protection against this Omicron strain.

Memory T cells are believed to contribute to the control of SARS-CoV-2 infection and replication, especially when serum neutralizing antibody titers are low^28,48–50^. Our experiments showed that i.n. delivered monovalent or bivalent ChAd-vectored vaccines elicited S protein-specific CD8^+^ T cells in the lung and BALF. The T_RM_ cell responses in the lung induced by the different ChAd vaccines were comparable because of the conservation of the immunodominant epitope in the S protein. Indeed, many T cell peptide epitopes are conserved among SARS-CoV-2 strains, and vaccine-induced T cells recognize the S peptides of antigenically distinct SARS-CoV-2 variants, including Omicron variants^51–55^. Our results are also consistent with human studies showing that the XBB sublineage is recognized by T cells following BA.5 bivalent mRNA vaccination despite extensive evasion of neutralizing antibody^56^.

All ChAd-SARS-CoV-2 vaccines prevented weight loss in K18-hACE2 mice induced by WA1/2020 or XBB.1.5 and conferred virological protection against WA1/2020 D614G and BQ.1.1 in nasal washes, nasal turbinates and lungs. Despite a relative paucity of neutralizing antibodies against XBB.1.5, lower levels of XBB.1.5 viral RNA were detected in the respiratory tract of ChAd-SARS-CoV-2-vaccinated animals. As mRNA vaccine-induced serum antibodies protect against antigenically distinct Omicron variants through Fc effector functions in the context of passive transfer^57,58^, this mechanism might explain ChAd-mediated control of XBB.1.5 infection. However, our passive transfer experiments suggest that although serum antibodies protect susceptible K18-hACE2 mice against challenge with homologous viruses, they do not efficiently protect against XBB.1.5 infection.

In the upper and lower respiratory tracts, we observed differences in protection by the matched and unmatched ChAd vaccines. Higher virus burden was seen in mice immunized with monovalent vaccines and challenged with heterologous viruses. Whereas infection in the lung was uniformly observed in XBB.1.5-challenged mice, viral burden and lung pathology were more limited in animals immunized with the more closely matched vaccine. Neutralizing antibodies are generally considered a key correlate of immune protection from SARS-CoV-2 infection^59^. Although our analyses revealed an association between total or neutralizing antibodies to WA1/2020 D614G or BQ.1.1 and lower viral burden in the lung, this relationship was not seen with XBB.1.5, suggesting that antibody levels were imperfect correlates of protection for this heterologous virus, at least in mice, possibly due to extensive immune evasion^11–13^. The bivalent ChAd vaccine conferred a small additional benefit against ancestral and antigenically distant variants, which is consistent with studies showing that boosting with bivalent mRNA vaccines confers more protection against some Omicron variants^42,60^.

Our CD8^+^ T cell depletion experiments showed that loss of memory CD8^+^ T cells compromises vaccine-mediated protection against XBB.1.5 infection. In the upper respiratory tract, memory CD8^+^ T cell responses showed a greater antiviral role during the early phase (day 3) of infection. Thus, vaccine-induced cross-reactive mucosal IgA and IgG responses or anamnestic B or CD4^+^ T cell responses appear insufficient to control XBB.1.5 infection in this compartment at this stage. By contrast, the antiviral effect of memory CD8^+^ T cells in the lung was seen at both 3 and 6 d.p.i. Although depletion of CD8^+^ T cells resulted in greater XBB.1.5 infection and pathology in the lung, this did not directly translate to effects on weight loss. This clinical phenotype might be explained by a threshold viral burden required for developing weight loss or a separate immunopathogenic contribution of CD8^+^ T cells in the lung in the context of SARS-CoV-2 infection. Indeed, *Rag1*^−/−^ mice, which lack B and T cells, show less lung disease after SARS-CoV infection than wild-type mice^61^.

A protective role for T cells in the setting of human SARS-CoV-2 infection or vaccination has been postulated^50,62^ in part because of the limited disease seen in some individuals with impaired antibody responses or receiving B cell-depleting drug therapies^63,64^. Our data are consistent with experiments in rhesus macaques showing that depletion of CD8^+^ T cells induced by natural SARS-CoV-2 infection results in higher viral loads after rechallenge^25^ and that CD8^+^ T cells induced by intramuscularly delivered Ad26.COV2.S contribute to virological control after challenge with SARS-CoV-2 B.1.617.2 (ref. ^26^). Our experiments are also supported by studies showing that adoptive transfer of infection-induced immune CD3^+^ T cells reduces SARS-CoV-2 burden in the hamster lung^65^ or that depletion of memory CD4^+^ and CD8^+^ T cells in K18-hACE2 mice immunized with a nucleocapsid-only vaccine impairs control of lung infection^66^. Our results differ from another study in K18-hACE2 mice, which showed that depletion of CD8^+^ T cells induced by the BNT162b2 mRNA vaccine had marginal impacts on virological control after challenge with the B.1.351 strain of SARS-CoV-2 (ref. ^49^). This difference in CD8^+^ T cell dependence of protection between models might be due to the antigenic distance between the vaccine and challenge virus, as described for influenza virus^67–69^, or differences in the route of immunization.

Syrian hamsters have been used to evaluate candidate SARS-CoV-2 vaccines. Whereas i.n. immunization with monovalent or bivalent ChAd-vectored vaccines elicited comparable antibody responses against ancestral WA1/2020 D614G virus, the bivalent vaccine induced greater responses against BA.5 and XBB.1.5. Furthermore, the bivalent ChAd vaccine conferred more protection against XBB.1.5, as demonstrated by lower viral burden in nasal turbinates, nasal washes and lungs, than the monovalent vaccine. Our data in hamsters validate the findings from mice and support the incorporation of more closely matched variant S in vaccine formulations to maximize protection against antigenically shifted SARS-CoV-2 variants.

We acknowledge limitations of our study. (1) Because of the terminal nature of the BAL procedure, we did not correlate pre-existing mucosal IgA and lung T cell responses with protection in our small animal models. (2) Although we evaluated the protection of i.n. delivered vaccines, effects on transmission were not studied. (3) Experiments with ChAd vaccines were performed as a single-dose vaccination. Considering that most individuals have some form of SARS-CoV-2 memory generated from repeated immunization and/or natural infection, the utility of i.n. delivered vaccines as boosters or in the context hybrid immunity requires investigation. (4) Although adenovirus-vectored COVID-19 vaccines delivered intramuscularly are associated with very rare thrombosis and thrombocytopenia^70^, these issues have not yet been reported with ChAd-SARS-CoV-2-S (iNCOVACC) and Ad5-nCoV-S (Convidecia Air) vaccines. The impact of different vaccine backbones and routes of administration on safety warrants investigation^71^.

Our studies provide evidence that an i.n. delivered bivalent ChAd vaccine can induce protective immunity that extends to antigenically shifted Omicron viruses even in the setting of limited serum neutralizing antibody responses, likely through effects of inhibitory CD8^+^ T cells, mucosally derived antibody and possibly CD4^+^ T cells. An updated formulation of this i.n. delivered vaccine holds promise for extending protection to strains that evade antibody responses generated by legacy vaccines.

## Methods

### Cells

African green monkey Vero-TMPRSS2 (ref. ^72^) and Vero-hACE2-TMPRSS2 (ref. ^73^) cells were cultured at 37 °C in Dulbecco’s modified Eagle medium (DMEM) supplemented with 10% fetal bovine serum (FBS), 10 mM HEPES (pH 7.3), 1 mM sodium pyruvate, 1× non-essential amino acids and 100 U ml^–1^ penicillin–streptomycin. Vero-TMPRSS2 cells were supplemented with 5 µg ml^–1^ blasticidin. Vero-hACE2-TMPRSS2 cells were supplemented with 10 µg ml^–1^ puromycin. All cells were routinely tested negative for *Mycoplasma* using a PCR-based assay.

### Viruses

The WA1/2020 strain with D614G substitution and BA.5.5 isolates were described previously^42,74^. The BQ.1, BF.7, XBB.1 and XBB.1.5 isolates were provided by A. Pekosz (Johns Hopkins University) and M. Suthar (Emory University) as part of the NIH SARS-CoV-2 Assessment of Viral Evolution Program^75^. All viruses were passaged once in Vero-TMPRSS2 cells, as described previously^76^, and were subjected to next-generation sequencing to confirm the identity of the virus and stability of the amino acid substitutions. All virus experiments were performed in approved biosafety level 3 facilities.

### Animals

Heterozygous K18-hACE2 C57BL/6J mice (strain 2B6.Cg-Tg(K18-ACE2)2Prlmn/J, 34860) were obtained from The Jackson Laboratory. Syrian hamsters were obtained from Charles River Laboratories and housed at Washington University. Animal studies were performed in accordance with the recommendations in the Guide for the Care and Use of Laboratory Animals of the National Institutes of Health. The protocols were approved by the Institutional Animal Care and Use Committee at the Washington University School of Medicine (assurance number A3381-01). Virus inoculations were performed under anesthesia that was induced and maintained with ketamine hydrochloride and xylazine (mice) or isoflurane (hamsters), and all efforts were made to minimize animal suffering. Sample sizes for animal experiments were determined on the basis of criteria set by the Institutional Animal Care and Use Committee. Experiments were neither randomized nor blinded. Mice were housed in groups of three to five, and hamsters were housed individually. Animals were maintained on a 12-h dark/12-h light cycle at an ambient room temperature of 21 °C (controlled within ±1 °C) and humidity of 50% (controlled within ±5%).

### Construction of chimpanzee adenovirus vectors

The ChAd-SARS-CoV-2-S replication-incompetent vector (simian Ad36) encoding the prefusion-stabilized SARS-CoV-2 S-2P and empty ChAd-Control vector were generated as described previously^15^. The ChAd-SARS-CoV-2-BA.5-S vector was constructed to express the prefusion-stabilized S glycoprotein of BA.5 (GenBank: QJQ84760; T19I, L24S, del25–27, del69–70, G142D, V213G, G339D, S371F, S373P, S375F, T376A, D405N, R408S, K417N, N440K, G446S, L452R, S477N, T478K, E484A, F486V, Q498R, N501Y, Y505H, D614G, H655Y, N679K, P681H, N764K, D796Y, Q954H and N969K) with six proline substitutions (F817P, A892P, A899P, A942P, K986P and V987P) and furin cleavage site substitutions (RRARS to GSASS, residues 682–686), as described elsewhere^77^. The ChAd-SARS-CoV-2-BA.5-S genome was rescued following transfection of the T-REx-293 cell line (Invitrogen, R710-07). Replication-incompetent ChAd-SARS-CoV-2-BA.5-S, ChAd-SARS-CoV-2-S and ChAd-Control vectors were scaled up in HEK-293 cells (ATCC, CRL-1573) and purified by CsCl density gradient ultracentrifugation. Viral particle concentrations were determined by spectrophotometry at 260 nm, as previously described^78^.

### Viral antigens

Recombinant Wuhan-1 and XBB.1 RBD proteins were a gift of D. Edwards (Moderna). Recombinant BA.5 and BQ.1.1 RBD proteins were produced in Expi293F cells (Thermo Fisher) by transfection of DNA using the ExpiFectamine 293 Transfection kit (Thermo Fisher). Supernatants were collected 3 d after transfection, and recombinant proteins were purified using Ni-NTA agarose (Thermo Fisher), buffer exchanged into PBS and concentrated using Amicon Ultracel centrifugal filters (EMD Millipore).

### Enzyme-linked immunosorbent assay (ELISA)

Purified recombinant Wuhan-1, BA.5, BQ.1.1 or XBB.1 RBD proteins were coated onto 96-well Maxisorp clear plates at 2 μg ml^–1^ in 50 mM Na_2_CO_3_ (pH 9.6; 50 μl) overnight at 4 °C. Coating buffers were aspirated, and wells were blocked with 200 μl of 1× PBS + 0.05% Tween 20 (PBST) + 2% bovine serum albumin + 0.02% NaN_3_ (blocking buffer) overnight at 4 °C. Sera or BALF samples were serially diluted in blocking buffer and added to the plates. Plates were incubated for 1 h at room temperature and washed three times with PBST, followed by the addition of 50 μl of 1:2,000 anti-mouse IgG–horseradish peroxidase (HRP; Southern Biotech, 1030-05) or anti-mouse IgA–biotin (Southern Biotech, 1040-08) and streptavidin–HRP (Vector Laboratories, SA-5004). Following a 1-h incubation at room temperature, plates were washed three times with PBST, and 50 μl of 1-Step Ultra TMB-ELISA was added (Thermo Fisher, 34028). Following a 2- to 5-min incubation, reactions were stopped with 50 μl of 2 M sulfuric acid. The absorbance of each well at 450 nm was determined using a microplate reader (BioTek) within 5 min of the addition of sulfuric acid. Endpoint serum titers were determined using a four-parameter logistic curve fit in GraphPad Prism version 10.0.

To measure hamster IgG responses, 96-well microtiter plates (Nunc MaxiSorp, Thermo Fisher Scientific) were coated with 100 µl of recombinant SARS-CoV-2 S protein (Wuhan-1 strain and BA.5) at a concentration of 1 µg ml^–1^ in PBS (Gibco) at 4 °C overnight; negative-control wells were coated with 1 µg ml^–1^ bovine serum albumin (Sigma). Plates were blocked for 1.5 h at room temperature with 280 µl of blocking solution (PBST (Sigma) and 10% FBS (Corning)). Sera were serially diluted in blocking solution, starting at a 1:100 dilution and incubated for 1.5 h at room temperature. Plates were washed three times with 1× PBST, and 50 µl of HRP-conjugated anti-hamster IgG (H + L; Southern Biotech, 6061-05) diluted 1:1,000 in blocking solution was added to all wells and incubated for 1 h at room temperature. Plates were washed three times with 1× PBST and three times with 1× PBS, and 50 µl of 1-step Ultra TMB-ELISA substrate solution (Thermo Fisher Scientific) was added to all wells. The reaction was stopped after 10 min using 50 µl of 1 N H_2_SO_4_, and the plates were analyzed at a wavelength of 450 nm using a microtiter plate reader (BioTek).

### Focus reduction neutralization test

Serial dilutions of mouse and hamster sera were incubated with 10^2^ f.f.u. of WA1/2020 D614G, BA.5, BF.7, BQ.1.1, XBB.1 or XBB.1.5 for 1 h at 37 °C. Antibody–virus complexes were added to Vero-TMPRSS2 cell monolayers in 96-well plates and incubated at 37 °C for 1 h. Subsequently, cells were overlaid with 1% (wt/vol) methylcellulose in MEM. Plates were collected 30 h (WA1/2020 D614G) or 52–66 h (Omicron strains) later by removing overlays and fixed with 4% paraformaldehyde in PBS for 20 min at room temperature. Plates were washed and sequentially incubated with an oligoclonal pool (SARS2-02, SARS2-08, SARS2-09, SARS2-10, SARS2-11, SARS2-13, SARS2-14, SARS2-17, SARS2-20, SARS2-26, SARS2-27, SARS2-28, SARS2-31, SARS2-38, SARS2-41, SARS2-42, SARS2-44, SARS2-49, SARS2-57, SARS2-62, SARS2-64, SARS2-65, SARS2-67 and SARS2-71 (ref. ^79^)) of mouse anti-S protein (including cross-reactive monoclonal antibodies to SARS-CoV) and HRP-conjugated goat anti-mouse IgG (Sigma, A8924; RRID: AB_258426) in PBS supplemented with 0.1% saponin and 0.1% bovine serum albumin. SARS-CoV-2-infected cell foci were visualized using TrueBlue peroxidase substrate (KPL) and quantitated on an ImmunoSpot microanalyzer (Cellular Technologies).

### Mouse experiments

Cohorts of 7- to 9-week-old female K18-hACE2 mice were immunized i.n. with 2 × 10^9^ viral particles of ChAd-SARS-CoV-2-S, ChAd-SARS-CoV-2-BA.5-S or ChAd-Bivalent vaccine (1:1 mixture of ChAd-SARS-CoV-2-S and ChAd-SARS-CoV-2-BA.5-S) in 50 µl of PBS. Animals were bled 4 weeks later to measure antibody responses. To evaluate protective activity, 8 weeks after immunization, K18-hACE2 mice were challenged i.n. with 10^4^ f.f.u. of WA1/2020 D614G, BQ.1.1 or XBB.1.5, and weights were recorded daily. Animals were killed at 6 d.p.i., and nasal washes, nasal turbinates and left lungs were collected for virological analyses. Right lung lobes were collected for pathological analyses. To assess whether ChAd-vectored vaccines alone induced inflammatory cytokine responses after i.n. delivery and the impact on early cytokine responses following SARS-CoV-2 challenge, separate sets of K18-hACE2 mice were immunized with monovalent or bivalent vaccines and challenged with XBB.1.5 using the same immunization scheme (Fig. 1b). Animals were killed 3 d after vaccination or 3 d.p.i. for cytokine and virological analyses.

For T cell analysis, the same vaccination scheme was applied, but animals were boosted with the same dose of homologous monovalent or bivalent vaccine 4 weeks later. Fourteen days later, the lungs and BALF samples were collected. To discriminate circulating from extravascular parenchymal immune cells, we administered 2 μg of APC/Fire750-labeled monoclonal anti-CD45 (BioLegend, clone 30-F11) to mice anesthetized with an overdose of ketamine and xylazine. After 3 min of labeling, mice were killed. BALF was collected through a plastic catheter clamped into the trachea using three separate lavages of 800 µl of PBS. Pooled BALF was centrifuged for 5 min at 600 × *g* at 4 °C, and the supernatant was stored at −20 °C for subsequent antibody analysis. Immune cells from BALF were resuspended in 2 ml of DMEM supplemented with 10% FBS and used for T cell phenotyping by spectral flow cytometry as described below. Lungs were collected in DMEM with 10% FBS on ice and minced with scissors and mesh through 70-µm cell strainers, and the cell suspensions were digested in HBSS containing 25 μg ml^–1^ DNase I (Roche, 11284932001) and 50 μg ml^–1^ Liberase (Roche, 5401119001) for 30 min at 37 °C. Subsequently, following hypotonic erythrocyte lysis, single cells were separated by passage through 70-µm cell strainers.

### S protein-specific T cell staining

For T cell analysis, single-cell suspensions were incubated with FcγR antibody (clone 93, BioLegend) to block non-specific antibody binding, followed by staining with a cocktail of labeled monoclonal antibodies, including Fixable Viability dye eFluor506, CD3e-BV711 (1:100, clone 145-2C11, BD Biosciences), CD8α-PerCP/Cyanine 5.5 (1:100, 53-6.7, BioLegend), CD4-BV785 (1:100, clone RM4-5, BioLegend), CD44-PE/Cyanine 7 (1:100, clone IM7, BioLegend), CD69-FITC (1:100, clone H1.2F3, BioLegend), CD103-PE (1:100, clone 2E7, BioLegend) and APC-labeled SARS-CoV-2 S-specific tetramer (MHC class I tetramer, residues 539–546, VNFNFNGL, H-2K^b^) for 60 min at room temperature. Cells were washed twice with FACS buffer and fixed with 2% paraformaldehyde for 20 min before data acquisition. Data were acquired on an Aurora (Cytek) spectral flow cytometer and analyzed with FlowJo v10 software.

### Passive serum transfer experiments

Seven- to 8-week-old female K18-hACE2 transgenic mice were administered 100 μl of pooled sera i.p. collected from mice 28 d after immunization with ChAd-Control, ChAd-SARS-CoV-2-S or ChAd-SARS-CoV-2-BA.5-S vaccine (Fig. 1b). One day later, mice were challenged with 50 μl of 10^4^ f.f.u. of WA1/2020 D614G or XBB.1.5 i.n. Daily weights were recorded, and lungs, nasal turbinates and nasal washes were collected at 6 d.p.i. for virological analysis. To assess serum antibody levels in the recipient mice after passive transfer, blood was collected 24 h after passive transfer. Serum levels of IgG targeting the RBD of Wuhan-1, BA-5 and XBB.1 were measured by ELISA, and neutralizing antibody titers were determined by focus reduction neutralization test.

### CD8^+^ T cell depletion experiments

In pilot experiments to assess the efficiency of depletion of CD8^+^ T cells, K18-hACE2 mice were immunized i.n. with a single dose (2 × 10^9^ viral particles) of ChAd-SARS-CoV-2-S vaccine. Mice were treated with anti-mouse CD8-depleting antibody (BioXell, clone YTS169.4) or with a sham isotype control antibody (rat IgG2b isotype, anti-Keyhole limpet hemocyanin, BioXell, BE0090) i.p. (250 µg in 100 µl of PBS) alone or in combination with i.n. administration (10 µg in 50 µl of PBS) using two different regimens: (1) i.p. administration beginning 1 d before vaccination and every 4 d thereafter plus i.n. administration 1 d before and 6 d after vaccination and also at days 26 and 29 (i.p./i.n., day –1 to day 30) or (2) i.p. and i.n. administration given 26 and 29 d after vaccination (i.p./i.n., days 26 and 29). On day 31 after immunization, spleens, BALF and lungs were analyzed for CD8^+^ T cells by flow cytometry.

In challenge experiments, cohorts of 7-week-old female K18-hACE2 mice were immunized i.n. with a single dose (2 × 10^9^ viral particles) of ChAd-Control or ChAd-SARS-CoV-2-S vaccine. Mice were treated with anti-mouse CD8-depleting antibody or with a sham isotype control antibody using the depletion regimens described above. Thirty days after vaccination, blood was collected for CD8^+^ T cell analysis by flow cytometry and for assessment of antibody responses by focus reduction neutralization test. Thirty-one days after vaccination, mice were challenged with 50 μl of 10^4^ f.f.u. of XBB.1.5 i.n. Daily weights were recorded. At 3 and 6 d.p.i., nasal washes, nasal turbinates and lungs were collected for viral burden analysis. At 6 d.p.i., BALF was collected for CD8^+^ T cell analysis.

### Hamster experiments

Cohorts of 9- to 10-week-old male Syrian golden hamsters were immunized i.n. with 1 × 10^10^ viral particles of ChAd-SARS-CoV-2-S (monovalent) or a 1:1 mixture of ChAd-SARS-CoV-2-S and ChAd-SARS-CoV-2-BA.5-S (bivalent) in 100 µl of PBS. Control animals received PBS intramuscularly. Animals were bled 3 weeks later to measure antibody responses. To evaluate protective activity, 5 weeks after immunization, hamsters were challenged i.n. with 10^5^ p.f.u. of XBB.1.5, and weights were recorded daily. Animals were killed at 3 d.p.i., and nasal washes, nasal turbinates and lungs were collected for virological analyses.

### Measurement of viral burden

Tissues from mice were weighed and homogenized with zirconia beads in a MagNA Lyser instrument (Roche Life Science) in 1 ml of DMEM supplemented with 2% heat-inactivated FBS. Tissue homogenates were clarified by centrifugation at 10,000 r.p.m. for 5 min and stored at −80 °C. RNA was extracted using a MagMax mirVana total RNA isolation kit (Thermo Fisher Scientific) on a Kingfisher Flex extraction robot (Thermo Fisher Scientific). RNA was reverse transcribed and amplified using a TaqMan RNA-to-CT 1-Step kit (Thermo Fisher Scientific). Reverse transcription was performed at 48 °C for 15 min followed by 2 min at 95 °C. Amplification was accomplished over 50 cycles as follows: 95 °C for 15 s and 60 °C for 1 min. Copies of SARS-CoV-2 *N* gene RNA in samples were determined using a published assay^80^. Tissues from hamsters were homogenized in 1 ml of DMEM, clarified by centrifugation (1,000 x *g* for 5 min) and used for viral titer analysis by quantitative real-time PCR using primers and probes targeting the *N* gene and by plaque assay. A nasal wash was also collected by flushing 1 ml of PBS with 0.1% bovine serum albumin into one nare and collecting the wash from the other. The nasal wash was clarified by centrifugation (2,000 × *g* for 10 min) and used for viral titer analysis. Plaque assays were performed on Vero-hACE2-hTRMPSS2 cells in 24-well plates. Lung tissue homogenates or nasal washes were serially diluted tenfold, starting at 1:10, in cell infection medium (DMEM + 2% FBS + 100 U ml^–1^ penicillin–streptomycin). Two hundred and fifty microliters of the diluted virus was added to a single well per dilution per sample. After 1 h at 37 °C, the inoculum was aspirated, the cells were washed with PBS, and a 1% methylcellulose overlay in MEM supplemented with 2% FBS was added. Ninety-six hours after virus inoculation, the cells were fixed with 10% formalin, and the monolayer was stained with crystal violet (0.5% (wt/vol) in 25% methanol in water) for 30 min at 20 °C. Plaque numbers were counted and used to calculate the p.f.u. per ml. Viral RNA was extracted from 100 µl of tissue or nasal wash samples using a MagNA Pure 24 Total NA Isolation kit on the MagNA Pure 24 system (Roche) and eluted with 50 µl of water. Four microliters of RNA was used for quantitative real-time PCR to detect and quantify expression of the SARS-CoV-2 *N* gene using a TaqMan RNA-to-CT 1-Step kit (Thermo Fisher Scientific), as previously described^81^, using the following primers and probes: primers: forward 5′-GACCCCAAAATCAGCGAAAT-3′ and reverse 5′-TCTGGTTACTGCCAGTTGAATCTG-3′; probe: 5′-ACCCCGCATTACGTTTGGTGGACC-3′; 5′Dye/3′Quencher 6-FAM/ZEN/IBFQ. Viral RNA was expressed as *N* gene copy numbers per mg for lung tissue and nasal turbinate homogenates or per ml for nasal washes and was based on a standard included in the assay created via in vitro transcription of a synthetic DNA molecule containing the target region of the *N* gene.

### Cytokine and chemokine measurements

Clarified lung homogenates were incubated with Triton X-100 (1% final concentration) for 1 h at room temperature to inactivate SARS-CoV-2. Homogenates were analyzed for cytokines and chemokines by Eve Technologies using their Mouse Cytokine Array/Chemokine Array 31-Plex (MD31) platform.

### Lung histology

Mouse lungs were inflated with 1 to 2 ml of 10% neutral buffered formalin using a 3-ml syringe after a catheter was inserted into the trachea. Lungs were then kept in fixative for 7 d. Tissues were embedded in paraffin, and sections were stained with hematoxylin and eosin. Images were captured using a Nanozoomer (Hamamatsu) at the Alafi Neuroimaging Core at Washington University. Lung tissue sections were evaluated and scored using two independent metrics. The first method used a blinded pathologist. The following scoring system was used to evaluate perivascular and peribronchial immune cell infiltration: 0, absent; 1, minor (small clusters of infiltrated cells); 2, moderate (medium-size clusters of infiltrated cells); 3, severe (large dense aggregates surrounding vessels). Interstitial lung disease was also evaluated using a composite H score^82–84^ based on the severity of interstitial inflammation, where 0 indicates no inflammation, 1 indicates mild immune cell infiltration in the septa, 2 indicates moderate immune cell infiltration with some thickened septa and the presence of inflammatory cells in the lumen, and 3 indicates severe immune infiltrates with thickened septa, air space consolidation and cellular aggregates in the lumen of the air space. The two scores were multiplied by the percentage of the lung affected, summed for each lung and divided by 100 to yield a composite score. The final normalized score was presented as 0–4. The second method used computational analysis. Stained lung images were analyzed quantitatively using ImageJ software^85^. Lung slides scanned by Nanozoomer were converted to ImageJ-compatible images following color threshold optimization and binary conversion. Areas with increased cellularity and tissue consolidation were differentiated from normal regions. The inflammatory and total areas were measured, and the percent inflammatory area was reported.

### Statistical and data analysis

Statistical significance was assigned when *P* values were <0.05 using GraphPad Prism version 10.0. Statistical tests (one-way ANOVA with a Dunnett’s post hoc test and one- or two-way ANOVA with a Tukey’s post hoc test), number of animals, median or mean values and comparison groups are indicated in the figure legends. Log-transformed viral RNA levels, serum antibody levels or cellular responses were analyzed by one-way ANOVA with multiple comparisons corrections. Data distribution was assumed to be normal, but this was not formally tested. Data collection and analysis were not performed blind to the conditions of the experiments. No data points were excluded from analysis.

### Reporting summary

Further information on research design is available in the Nature Portfolio Reporting Summary linked to this article.

## Online content

Any methods, additional references, Nature Portfolio reporting summaries, source data, extended data, supplementary information, acknowledgements, peer review information; details of author contributions and competing interests; and statements of data and code availability are available at 10.1038/s41590-024-01743-x.

### Supplementary information

Reporting Summary

### Source data

Source Data Fig. 1 Experimental data for statistical analysis and graphs.

Source Data Fig. 2 Experimental data for statistical analysis and graphs.

Source Data Fig. 3 Experimental data for statistical analysis and graphs.

Source Data Fig. 4 Experimental data for statistical analysis and graphs.

Source Data Fig. 5 Experimental data for statistical analysis and graphs.

Source Data Fig. 6 Experimental data for statistical analysis and graphs.

Source Data Fig. 7 Experimental data for statistical analysis and graphs.

Source Data Fig. 8 Experimental data for statistical analysis and graphs.

Source Data Extended Data Fig 1 Experimental data for statistical analysis and graphs.

Source Data Extended Data Fig. 2 Experimental data for statistical analysis and graphs.

Source Data Extended Data Fig. 4 Experimental data for statistical analysis and graphs.

Source Data Extended Data Fig. 5 Experimental data for statistical analysis and graphs.

Source Data Extended Data Fig. 6 Experimental data for statistical analysis and graphs.

Source Data Extended Data Fig. 7 Experimental data for statistical analysis and graphs.

Source Data Extended Data Fig. 8 Experimental data for statistical analysis and graphs.

## Data Availability

All data supporting the findings of this study are available within the paper and its supporting information, and all reagents are available through a material transfer agreement. Source data are provided with this paper. Any additional information related to the study is available from the corresponding author upon request.
