# Author Correction: Mucosal vaccine-induced cross-reactive CD8^+^ T cells protect against SARS-CoV-2 XBB.1.5 respiratory tract infection

**DOI:** 10.1038/s41590-024-01781-5

**Published:** 2024-02-15

**Authors:** Baoling Ying, Tamarand L. Darling, Pritesh Desai, Chieh-Yu Liang, Igor P. Dmitriev, Nadia Soudani, Traci Bricker, Elena A. Kashentseva, Houda Harastani, Saravanan Raju, Meizi Liu, Aaron G. Schmidt, David T. Curiel, Adrianus C. M. Boon, Michael S. Diamond

**Affiliations:** 1 Department of Medicine, Washington University School of Medicine, St. Louis, MO USA; 2 Department of Pathology & Immunology, Washington University School of Medicine, St. Louis, MO USA; 3 Department of Radiation Oncology, Washington University School of Medicine, St. Louis, MO USA; 4 Ragon Institute of Massachusetts General Hospital, Massachusetts Institute of Technology and Harvard University, Cambridge, MA USA; 5 Department of Microbiology, Harvard Medical School, Boston, MA USA; 6 Department of Molecular Microbiology, Washington University School of Medicine, St. Louis, MO USA; 7 Andrew M. and Jane M. Bursky Center for Human Immunology and Immunotherapy Programs, Washington University School of Medicine, St. Louis, MO USA; 8 Center for Vaccines and Immunity to Microbial Pathogens, Washington University School of Medicine, St. Louis, MO USA

**Keywords:** RNA vaccines, Viral infection, Immunological memory, SARS-CoV-2

Correction to: *Nature Immunology* 10.1038/s41590-024-01743-x, published online 9 February 2024.

In the version of this article initially published, due to a mistake in figure assembly, the images shown in the “Naive” column of Fig. 5c,e were duplicates of Fig. 5a. The error was in presentation only. The figure has been updated in the HTML and PDF versions of the article.

