# Supplementary material for: Mucosal vaccine-induced cross-reactive CD8+ T cells protect against SARS-CoV-2 XBB.1.5 respiratory tract infection
Source: Nat Immunol. 2024 Feb 9;25(3):537–51. doi: 10.1038/s41590-024-01743-x (PMC10907304; doi:10.1038/s41590-024-01743-x)
Supplement: Supplementary file 1 — Reporting Summary [file 41590_2024_1743_MOESM1_ESM.pdf]

nature portfolio

Corresponding author(s): Michael Diamond

Last updated by author(s): Dec 8, 2023

Reporting Summary

Nature Portfolio wishes to improve the reproducibility of the work that we publish. This form provides structure for consistency and transparency in reporting. For further information on Nature Portfolio policies, see our Editorial Policies and the Editorial Policy Checklist.

Statistics

For all statistical analyses, confirm that the following items are present in the figure legend, table legend, main text, or Methods section.

n/a	Confirmed	
☐	☒	The exact sample size (*n*) for each experimental group/condition, given as a discrete number and unit of measurement
☐	☒	A statement on whether measurements were taken from distinct samples or whether the same sample was measured repeatedly
☐	☒	The statistical test(s) used AND whether they are one- or two-sided<br>*Only common tests should be described solely by name; describe more complex techniques in the Methods section.*
☒	☐	A description of all covariates tested
☐	☒	A description of any assumptions or corrections, such as tests of normality and adjustment for multiple comparisons
☒	☐	A full description of the statistical parameters including central tendency (e.g. means) or other basic estimates (e.g. regression coefficient) AND variation (e.g. standard deviation) or associated estimates of uncertainty (e.g. confidence intervals)
☐	☒	For null hypothesis testing, the test statistic (e.g. *F*, *t*, *r*) with confidence intervals, effect sizes, degrees of freedom and *P* value noted<br>*Give P values as exact values whenever suitable.*
☒	☐	For Bayesian analysis, information on the choice of priors and Markov chain Monte Carlo settings
☒	☐	For hierarchical and complex designs, identification of the appropriate level for tests and full reporting of outcomes
☒	☐	Estimates of effect sizes (e.g. Cohen's *d*, Pearson's *r*), indicating how they were calculated

Our web collection on statistics for biologists contains articles on many of the points above.

Software and code

Policy information about availability of computer code

Data collection	No software was used in this study to collect data
Data analysis	Prism 10.0 was used to perform all statistical analysis. Flow cytometry data were acquired on an Aurora (Cytek) spectral flow cytometer and analyzed in FlowJo v10 software. The stained lung staining images were analyzed quantitativety using Image J software

For manuscripts utilizing custom algorithms or software that are central to the research but not yet described in published literature, software must be made available to editors and reviewers. We strongly encourage code deposition in a community repository (e.g. GitHub). See the Nature Portfolio guidelines for submitting code & software for further information.

Data

Policy information about availability of data

All manuscripts must include a data availability statement. This statement should provide the following information, where applicable:

- Accession codes, unique identifiers, or web links for publicly available datasets
- A description of any restrictions on data availability
- For clinical datasets or third party data, please ensure that the statement adheres to our policy

All data supporting the findings of this study are available within the paper, its Supplemental or Extended Data files, or Source files. Any additional information related to the study is available from the corresponding author upon request. All reagents are available through a Material Transfer Agreement.

Field-specific reporting

Please select the one below that is the best fit for your research. If you are not sure, read the appropriate sections before making your selection.

☒ Life sciences ☐ Behavioural & social sciences ☐ Ecological, evolutionary & environmental sciences

For a reference copy of the document with all sections, see nature.com/documents/nr-reporting-summary-flat.pdf

Life sciences study design

All studies must disclose on these points even when the disclosure is negative.

Sample size	No sample sizes were chosen a priori but instead estimated based on prior knowledge of anticipated experimental differences among groups. All experiments with statistical analysis were repeated at least two independent times, each with multiple technical replicates. Experimental size of animal cohorts was determined based on prior experience performing studies in mice.
Data exclusions	No data was excluded.
Replication	All experiments had multiple biological and/or technical replicates and are indicated the Figure legends.
Randomization	For animal studies, mice were randomly assigned from large batches obtained from the vendor to different experimental groups in an age-matched distribution.
Blinding	No blinding was performed although several key studies were performed independently by multiple members of the group

Reporting for specific materials, systems and methods

We require information from authors about some types of materials, experimental systems and methods used in many studies. Here, indicate whether each material, system or method listed is relevant to your study. If you are not sure if a list item applies to your research, read the appropriate section before selecting a response.

Materials & experimental systems

n/a	Involved in the study	
☐	☒	Antibodies
☐	☒	Eukaryotic cell lines
☒	☐	Palaeontology and archaeology
☐	☒	Animals and other organisms
☒	☐	Human research participants
☒	☐	Clinical data
☒	☐	Dual use research of concern

Methods

n/a	Involved in the study	
☒	☐	ChIP-seq
☐	☒	Flow cytometry
☒	☐	MRI-based neuroimaging

Antibodies

Antibodies used

Mice were treated with anti-mouse CD8 depleting antibody (BioXell, clone YTS169.4, Cat # BE0117) or with a sham isotype-control antibody (rat IgG2b isotype, anti-Keyhole limpet hemocyanin, BioXell, # BE0090) administered via IP route (250 µg in 100 µL PBS).

For T cell analysis, single cell suspensions were incubated with FcyR antibody (clone 93, BioLegend, Cat # 101335) to block non-specific antibody binding, followed by staining with a cocktail of labeled mAbs including Fixable Viability dye eFluor506, CD3e-BV711 (1:100, clone:145-2C11, BD Biosciences, Cat # 563123), CD8α-PerCP/Cyanine 5.5 (1:100, 53–6.7, BioLegend, Cat # 100732), CD4-BV785 (1:100, clone: RM4-5, BioLegend, Cat # 100551), CD44-PE/Cyanine 7 (1:100, clone: IM7, BioLegend, Cat # 103007), CD69-FITC (1:100, clone: H1.2F3, BioLegend, Cat # 104505), CD103-PE (1:100, clone: 2E7, BioLegend, Cat # 121406) and APC-labeled SARS-CoV-2 S- specific tetramer (MHC class I tetramer, residues 539–546, VNFNFNGL, H-2K(B).

To discriminate circulating from extravascular parenchymal immune cells, we administered 2 ug of APC/Fire750-labeled anti-CD45 mAb (Biolegend, clone 30-F11, Cat # 103154) to mice.

For ELISAa: Hamster IgG: HRP-conjugated anti-hamster IgG(H+L) antibody (Southern Biotech Cat. #6061-05) diluted 1:1000 in blocking solution; Mouse IgG and IgA: Addition of 50 ⍰L of 1:2000 anti-mouse IgG-HRP (Southern Biotech, Cat. #1030-05), or anti-mouse IgA-biotin (Southern Biotech, Cat. #1040-08) and then streptavidin-HRP (Vector laboratories, Cat. SA-5004).

For FRNT assay: Plates were washed and sequentially incubated with an oligoclonal pool (SARS2-02, -08, -09, -10, -11, -13, -14, -17, -20, -26, -27, -28, -31, -38, -41, -42, -44, -49, , -57, -62, -64, -65, -67, and -71 (all made by Diamond laboratory) of anti-spike murine antibodies (including cross-reactive mAbs against SARS-CoV and HRP-conjugated goat anti-mouse IgG (Sigma Cat # A8924, RRID: AB_258426) in PBS.

Validation	All primary Abs were validated using purified viral proteins by ELISA or transfected/infected cells by flow cytometry. All secondary antibodies were validated against indicated proteins by the manufacturer per their associated Data Sheets.

Eukaryotic cell lines

Policy information about cell lines

Cell line source(s)	African green monkey Vero-TMPRSS2 and Vero-hACE2-TMPRSS2 cells were generated in the Diamond laboratory. HEK-293 cells were purchased commercially (ATCC, CRL-1573). Expi293F cells were purchased commercially (ThermoFisher, Cat # A14527)
Authentication	These cells grew as expected and propagated virus as expected. Also, we confirmed expression of specific transgenes using antibodies and flow cytometry.
Mycoplasma contamination	All cell lines are routinely tested each month and were negative for mycoplasma.
Commonly misidentified lines (See ICLAC register)	This study did not involve any commonly misidentified cell lines.

Animals and other organisms

Policy information about studies involving animals; ARRIVE guidelines recommended for reporting animal research

Laboratory animals	Seven to nine-week-old female heterozygous K18-hACE2 C57BL/6J mice (strain: 2B6.Cg-Tg(K18-ACE2)2Prlmn/J, Cat # 34860) were obtained from The Jackson Laboratory and used in immunization studies. Nine to ten-week-old male Syrian hamsters were obtained from Charles River Laboratories and housed at Washington University. Mice were housed in groups of 3 to 5. Hamsters were housed by themselves in groups of 1. Photoperiod = 12 hr on:12 hr off dark/light cycle. Ambient animal room temperature is 70° F, controlled within ±2° and room humidity is 50%, controlled within ±5%.
Wild animals	No wild animals were used in this study.
Field-collected samples	No field collected samples were used in this study.
Ethics oversight	All experiments were conducted with approval of the Institutional Animal Care and Use Committee at the Washington University School of Medicine (Assurance number A3381-01)

Note that full information on the approval of the study protocol must also be provided in the manuscript.

Flow Cytometry

Plots

Confirm that:

☒ The axis labels state the marker and fluorochrome used (e.g. CD4-FITC).

☒ The axis scales are clearly visible. Include numbers along axes only for bottom left plot of group (a 'group' is an analysis of identical markers).

☒ All plots are contour plots with outliers or pseudocolor plots.

☒ A numerical value for number of cells or percentage (with statistics) is provided.

Methodology

Sample preparation	For T cell analysis, single cell suspensions were incubated with FcγR antibody (clone 93, BioLegend) to block non-specific antibody binding, followed by staining with a cocktail of labeled mAbs including Fixable Viability dye eFluor506, CD3e-BV711 (1:100, clone:145-2C11, BD Biosciences), CD8α-PerCP/Cyanine 5.5 (1:100, 53–6.7, BioLegend), CD4-BV785 (1:100, clone: RM4-5, BioLegend), CD44-PE/Cyanine 7 (1:100, clone: IM7, BioLegend), CD69-FITC (1:100, clone: H1.2F3, BioLegend), CD103-PE (1:100, clone: 2E7, BioLegend) and APC-labeled SARS-CoV-2 S- specific tetramer (MHC class I tetramer, residues 539–546, VNFNFNGL, H-2K(B) for 60 min at room temperature. Cells were washed twice with FACS buffer, fixed with 2% paraformaldehyde (PFA) for 20 min prior to data acquisition. Data were acquired on an Aurora (Cytek) spectral flow cytometer and analyzed in FlowJo v10 software.
Instrument	Aurora (Cytek)
Software	FlowJo v10
Cell population abundance	Cells were greater than 15% of all total events (from a spleen)
Gating strategy	CD8+ T cells were gated as live, singlets, CD3+ and CD8+ cells, then tetramer+CD44+ and CD103+CD69+ (see Extended Data Fig 3)

☒ Tick this box to confirm that a figure exemplifying the gating strategy is provided in the Supplementary Information.
